# Independent and combined effects of improved water, sanitation, and hygiene (WASH) and improved complementary feeding on early neurodevelopment among children born to HIV-negative mothers in rural Zimbabwe: Substudy of a cluster-randomized trial

**DOI:** 10.1371/journal.pmed.1002766

**Published:** 2019-03-21

**Authors:** Melissa J. Gladstone, Jaya Chandna, Gwendoline Kandawasvika, Robert Ntozini, Florence D. Majo, Naume V. Tavengwa, Mduduzi N. N. Mbuya, Goldberg T. Mangwadu, Ancikaria Chigumira, Cynthia M. Chasokela, Lawrence H. Moulton, Rebecca J. Stoltzfus, Jean H. Humphrey, Andrew J. Prendergast, for the SHINE Trial Team

**Affiliations:** 1 Department of Women and Children’s Health, Institute of Translational Medicine, University of Liverpool, Liverpool, United Kingdom; 2 Zvitambo Institute for Maternal and Child Health Research, Harare, Zimbabwe; 3 University of Zimbabwe, Harare, Zimbabwe; 4 Global Alliance for Improved Nutrition, Washington, District of Columbia, United States of America; 5 Ministry of Health and Child Care, Government of Zimbabwe, Harare, Zimbabwe; 6 Department of International Health, Johns Hopkins Bloomberg School of Public Health, Baltimore, Maryland, United States of America; 7 Division of Nutritional Sciences, Cornell University, Ithaca, New York, United States of America; 8 Blizard Institute, Queen Mary University of London, London, United Kingdom; Makerere University Medical School, UGANDA

## Abstract

**Background:**

Globally, nearly 250 million children (43% of all children under 5 years of age) are at risk of compromised neurodevelopment due to poverty, stunting, and lack of stimulation. We tested the independent and combined effects of improved water, sanitation, and hygiene (WASH) and improved infant and young child feeding (IYCF) on early child development (ECD) among children enrolled in the Sanitation Hygiene Infant Nutrition Efficacy (SHINE) trial in rural Zimbabwe.

**Methods and findings:**

SHINE was a cluster-randomized community-based 2×2 factorial trial. A total of 5,280 pregnant women were enrolled from 211 clusters (defined as the catchment area of 1–4 village health workers [VHWs] employed by the Zimbabwean Ministry of Health and Child Care). Clusters were randomly allocated to standard of care, IYCF (20 g of small-quantity lipid-based nutrient supplement per day from age 6 to 18 months plus complementary feeding counseling), WASH (ventilated improved pit latrine, handwashing stations, chlorine, liquid soap, and play yard), and WASH + IYCF. Primary outcomes were child length-for-age *Z*-score and hemoglobin concentration at 18 months of age. Children who completed the 18-month visit and turned 2 years (102–112 weeks) between March 1, 2016, and April 30, 2017, were eligible for the ECD substudy. We prespecified that primary inferences would be drawn from findings of children born to HIV-negative mothers; these results are presented in this paper. A total of 1,655 HIV-unexposed children (64% of those eligible) were recruited into the ECD substudy from 206 clusters and evaluated for ECD at 2 years of age using the Malawi Developmental Assessment Tool (MDAT) to assess gross motor, fine motor, language, and social skills; the MacArthur–Bates Communicative Development Inventories (CDI) to assess vocabulary and grammar; the A-not-B test to assess object permanence; and a self-control task. Outcomes were analyzed in the intention-to-treat population. For all ECD outcomes, there was not a statistical interaction between the IYCF and WASH interventions, so we estimated the effects of the interventions by comparing the 2 IYCF groups with the 2 non-IYCF groups and the 2 WASH groups with the 2 non-WASH groups. The mean (95% CI) total MDAT score was modestly higher in the IYCF groups compared to the non-IYCF groups in unadjusted analysis: 1.35 (0.24, 2.46; *p =* 0.017); this difference did not persist in adjusted analysis: 0.79 (−0.22, 1.68; *p =* 0.057). There was no evidence of impact of the IYCF intervention on the CDI, A-not-B, or self-control tests. Among children in the WASH groups compared to those in the non-WASH groups, mean scores were not different for the MDAT, A-not-B, or self-control tests; mean CDI score was not different in unadjusted analysis (0.99 [95% CI −1.18, 3.17]) but was higher in children in the WASH groups in adjusted analysis (1.81 [0.01, 3.61]). The main limitation of the study was the specific time window for substudy recruitment, meaning not all children from the main trial were enrolled.

**Conclusions:**

We found little evidence that the IYCF and WASH interventions implemented in SHINE caused clinically important improvements in child development at 2 years of age. Interventions that directly target neurodevelopment (e.g., early stimulation) or that more comprehensively address the multifactorial nature of neurodevelopment may be required to support healthy development of vulnerable children.

**Trial registration:**

ClinicalTrials.gov NCT01824940

## Abstract 2

In this substudy from a 2x2 factorial cluster-randomized trial, Melissa Gladstone and colleagues assess whether improved WASH and/or feeding provide a benefit to early child development.

## Author summary

**Why was this study done?:**

- Some 43% of children globally fail to reach their full developmental potential due to stunting and poverty.
- Current evidence shows that improved nutrition has a modest effect on early child development.
- Improving water, sanitation, and hygiene (WASH) may plausibly benefit neurodevelopment through reduced illness and improved gut health (through improving nutrient absorption and optimizing gut–brain communication).

**What did the researchers do and find?:**

- The Sanitation Hygiene Infant Nutrition Efficacy (SHINE) trial tested the individual and combined effects of improved complementary feeding (provision of a small quantity of lipid-based nutrient supplement from 6 to 18 months of age, with complementary feeding counseling) and improved household WASH (provision of a pit latrine, handwashing stations, soap, chlorine, and hygiene counseling) on early child development at 24 months.
- In all, 1,655 children born to HIV-negative women were assessed for gross motor, fine motor, language, cognitive, and social development using tools that were designed and adapted for rural Zimbabwe.
- We found little evidence that the complementary feeding or WASH interventions tested improved child neurodevelopment at 2 years of age.

**What do these findings mean?:**

- Complementary feeding and WASH interventions (as described above) may not have a clinically significant impact on child neurodevelopment.
- More holistic approaches and interventions that explicitly target early child development may be needed to substantially impact child neurodevelopment.

## Introduction

Globally, nearly 250 million children (43% of all children under 5 years of age) are at risk of compromised neurodevelopment due to poverty, stunting, and lack of stimulation [1]. Stunting has now been inextricably linked to poor early child development (ECD) [2] and affects 150 million children globally [3,4]. Although studies have demonstrated some improvements in ECD related to improved feeding practices, these studies have not demonstrated as much effect as hoped [5]. To address this “silent emergency” of compromised developmental potential in children [6], the Global Strategy for Women’s, Children’s and Adolescents’ Health (2016–2030) [7] and the recent Nurturing Care Framework [8]—promoted by international organizations including the World Health Organization, the World Bank, and UNICEF—are calling for an urgent scale-up of explicit ECD interventions, such as age-appropriate stimulation, responsive care, and increased access to high-quality pre-primary education. In parallel, the World Health Assembly has called for a 40% reduction in stunting by 2025 [9]. It is clear that action toward reducing the network of underlying factors that indirectly cause poor developmental outcomes is necessary. Predominant among these factors are nutritionally inadequate infant diets, and low and inequitable coverage of clean water, sanitation, and hygiene.

Among nutrition interventions, improved breastfeeding practices including early initiation [10], exclusive breastfeeding to age 6 months, increased duration of breastfeeding, and continued breastfeeding to age 24 months [11] have been shown to reduce diarrhea and child mortality, and improve educational attainment and adult income [12]. This is likely due to direct effects of nutrient provision on brain development as well as indirect effects of nutrition on physical growth, motor development, and physical activity [13]. Despite this, currently only 50% of children are breastfeed in the first hour after birth, and only 37% are exclusively breastfed [11]. Improved complementary feeding can reduce stunting [14], which is one of the strongest risk factors for poor ECD [2]; furthermore, long-term follow-up of randomized trials demonstrates that improving the nutritional adequacy of children’s diets between age 6 months and 3 years improves adult cognition and economic productivity [15].

Water, sanitation, and hygiene (WASH) interventions may also plausibly improve ECD. In a randomized trial in Pakistan, children whose households had received a 9-month intensive handwashing promotion during the first 30 months of life, which reduced diarrhea during that period but not subsequently, had higher global developmental quotients at age 5–7 years in comparison to control children, despite similar anthropometric measurements in children across the groups [16]. WASH interventions may impact ECD through several interlinked pathways. First, sanitation and handwashing with soap can reduce childhood diarrheal disease, which in some studies has been linked to poor childhood cognition and school performance [17,18], although this effect does not remain once stunting is taken into account [19,20]. Second, WASH may plausibly improve cognition by preventing environmental enteric dysfunction (EED), which may be an underlying cause of stunting [21]. EED is a disorder of the small intestine that is virtually ubiquitous among people living in conditions of poor sanitation and hygiene and is characterized by villous atrophy, increased permeability, malabsorption, and inflammation [22]. Third, WASH may modulate the composition and function of the gut microbiota, thereby influencing brain development through the microbiota–gut–brain axis [23]. Finally, EED is accompanied by systemic inflammation, which may directly impair neurodevelopment [24], and indirectly drive anemia through reduced erythropoiesis and hepcidin-mediated iron deficiency. Iron deficiency directly compromises brain development through its role in myelination, neurotransmission, and protein expression [25], and anemia causes listlessness. Similarly, being sick with diarrhea is likely to also impact children’s ability or willingness to engage in learning or active play. This resulting listlessness and lack of interest can then lead to reduced caregiver–child interaction and the capacity for children to engage in stimulating interactions and positive exploratory play [26].

It is therefore plausible that combining improved WASH and improved infant and young child feeding (IYCF) could impact ECD. The objective of this substudy within the Sanitation Hygiene Infant Nutrition Efficacy (SHINE) trial [27] was to test this hypothesis by evaluating the independent and combined effects of improved WASH and improved IYCF on ECD.

## Methods

### The SHINE trial

The design and methods of SHINE have been previously described [27]; the full protocol and statistical analysis plan are at https://osf.io/w93hy. Briefly, SHINE was a cluster-randomized community-based 2×2 factorial trial testing the independent and combined effects of a WASH intervention and an IYCF intervention on linear growth and hemoglobin at 18 months of age [27]. The study area comprised 2 rural districts of central Zimbabwe, which were divided into 212 clusters, defined as the catchment area of 1–4 village health workers (VHWs) employed by the Zimbabwean Ministry of Health and Child Care. Clusters were randomly allocated to 1 of 4 treatment arms (standard of care [SOC] alone, WASH, IYCF, or WASH + IYCF) at a public event using highly constrained randomization, which achieved balance across arms on 14 measures of geography, demography, water access, and sanitation coverage [28] (described more fully in S1 Text). Due to the nature of the interventions, masking was not possible. Between November 2012 and March 2015, VHWs identified pregnancies through prospective surveillance; women were eligible if they permanently resided in 1 of the rural study clusters, were confirmed pregnant (<14 gestational weeks), and provided written informed consent. Over the recruitment period, the cutoff of gestational age for recruitment eligibility was increased to 18 weeks (August 22, 2013), 24 weeks (January 3, 2014), and any time prior to parturition (October 20, 2014), through trial protocol amendments, to maximize recruitment. Recruitment took place in 211 of the 212 clusters.

### Intervention delivery

All women were scheduled to receive 15 VHW visits between enrollment and 12 months postpartum (approximately 1 visit/month). Interventions were informed by extensive formative research and piloting [27,29,30]. Participatory behavior change interventions delivered during these visits were arm-specific and grounded in behavior change theory [27]. The IYCF intervention was based on formative research to identify and target cultural barriers [31]. The WASH intervention was based on the model of planned, motivated, and habitual hygiene behavior and was designed to invoke motivating emotions for hygiene and nurture [32]. At each visit, previous information was reviewed before introducing new information to create a sequenced integrated longitudinal intervention. Between 13 and 17 months postpartum, VHWs undertook monthly visits to provide routine care, deliver intervention supplies, and provide informal reminders to practice relevant behaviors, but formal modules were not delivered. At 18 months postpartum, an intervention review module was delivered to mothers in all trial arms.

VHW supervisors assessed timing and fidelity of implementation during scheduled visits and spot-checks (conducted every 3 months, or more often if VHW performance was not optimal). The content of VHW visits and the commodities provided were arm-specific.

#### SOC

VHWs delivered a breastfeeding intervention during 4 visits between late pregnancy and 3 months postpartum [27], and promoted family planning, immunization, and prevention of mother-to-child-transmission (PMTCT) of HIV.

#### WASH

VHWs delivered all SOC interventions plus modules promoting safe disposal of feces, handwashing with soap, protection of infants from geophagia and animal feces ingestion [27,29], chlorination of drinking water, and hygienic handling and preparation of complementary food. A Blair ventilated improved pit latrine was constructed within 6 weeks of enrollment by builders hired from the study communities; the study provided all materials and labor. Two handwashing stations were installed by 32 weeks’ gestation with monthly delivery of liquid soap until 18 months postpartum. A plastic baby mat and play yard were delivered at 2 months postpartum to protect children from geophagia. Chlorination solution (WaterGuard, Nelspot, Zimbabwe) was delivered monthly between 4 and 18 months postpartum for water treatment [27].

#### IYCF

VHWs delivered all SOC interventions plus modules promoting nutrient-dense, diverse infant diets using locally available foods processed to facilitate mastication and swallowing, and frequent responsive feeding during illness. Small-quantity lipid-based nutrient supplement (SQ-LNS; Nutriset, Malaumay, France) was provided from 6 to 18 months postpartum; caregivers added a 20-g sachet daily to complementary food [27,30]. SQ-LNS was an supplement and was not intended to replace other food.

#### WASH + IYCF

VHWs delivered all SOC, WASH, and IYCF interventions.

After trial completion, a latrine was constructed for families in the SOC and IYCF arms.

### Data collection

Research nurses, separate from the intervention teams, made home visits at baseline (2 weeks after consent), at 32 weeks’ gestation, and at 1, 3, 6, 12, and 18 months postpartum to assess maternal and household characteristics and trial outcomes. At baseline, mothers had weight, mid-upper arm circumference (MUAC), and hemoglobin (Hemocue, Ängelholm, Sweden) measured, and were tested for HIV using a rapid test algorithm. HIV-positive women were urged to seek immediate antenatal care for PMTCT interventions. The following baseline indices were assessed: household minimum dietary diversity, food insecurity (Coping Strategies Index), household wealth (asset ownership index) [33], and maternal capabilities (perceived physical health, mental health, stress, social support, decision-making autonomy, gender norms attitudes, time use, and mothering self-efficacy), as detailed in the trial design paper [27].

Infant birth date, weight, and delivery details were transcribed from health records. The trial provided Tanita BD-590 infant scales to all health institutions in the study area and conducted training. Gestational age at delivery (prematurity) was calculated from the date of the last menstrual period. Infant weight, length, head circumference, and MUAC were measured at every postnatal visit. Children with acute malnutrition or illness were referred to local clinics. Mothers testing HIV-negative at baseline were retested at 32 weeks’ gestation; those testing HIV-negative during pregnancy were retested at 18 months postpartum.

Intervention uptake was assessed at all visits and is reported here, as pre-specified, for the 12-month postnatal visit. Nurses assessed WASH-related behaviors through maternal report (open defecation among household members, treatment of drinking water, disposal of nappy water, and child geophagia) and observation of the latrine (type of latrine, whether the path to latrine was trodden, whether the latrine was used for storage; and whether the latrine was shared with other households), handwashing station (presence of Tippy Taps and whether they were filled with soap and water), and play yard (visible cleanliness). Nurses assessed IYCF behaviors through maternal report of whether the child was still breastfeeding; the mother’s understanding of how to feed a child during illness; 24-hour recall of infant minimum dietary diversity and consumption of iron-rich, animal-source, and vitamin-A-rich foods; and 24-hour recall of infant SQ-LNS consumption.

### ECD substudy

#### Population and recruitment

The ECD substudy was conducted, among a subgroup of children enrolled in SHINE, to assess the impact of the SHINE interventions on child development at 24 months of age. No additional interventions were provided after 18 months. Children were eligible for the ECD substudy if they had the trial primary outcomes (linear growth and hemoglobin) measured at 18 months of age, and turned 2 years of age (allowable range 102–112 weeks) between March 2016 and April 30, 2017. The substudy initiation date was staggered across research sites from March 1 to March 15, 2016, so that the first visits could be directly supervised by the trial psychologist (JC). Written informed consent for the ECD substudy was obtained where possible during the 18-month SHINE visit. For those who had already had their 18-month visit, families were contacted through their VHW, by phone call, or through a home visit to establish their interest in joining the ECD substudy; written informed consent was then obtained prior to conducting the ECD assessment. We compared baseline characteristics across the 4 treatment arms among 3 groups of children who completed the trial 18-month visit: those who were not eligible for the ECD substudy, those who were eligible and recruited into the ECD substudy, and those who were eligible but not recruited into the ECD substudy.

All assessments were conducted in the home over a period of 2–3 hours by 1 of 11 SHINE research nurses who completed 3 weeks of residential training in ECD assessments. All children enrolled into the ECD substudy were assessed, but children who scored “moderate to severe” on the Washington screen for disability [34] were excluded from analysis and referred for appropriate services.

#### Assessment tools

All assessment tools used for measuring outcomes were directly observed and were done on 1 occasion, with the child at the age of 24 months.

The Malawi Developmental Assessment Tool (MDAT) measures child development in 4 domains: (1) gross motor coordination (36 items), (2) fine motor coordination (36 items), (3) language (36 items), and (4) social (30 items), with a total of 138 items. The fine motor, language, and social domains also measure components of cognitive development [35]. Following translation, back translation, and piloting, minor adaptations were made to kit items, particularly those that the child had to name or identify as part of the language assessment.

The MacArthur–Bates Communicative Development Inventories (CDI) [36] assesses child language according to maternal report, including a vocabulary and grammar checklist. The test was specifically adapted for Shona speakers using a detailed protocol approved by the CDI team [37,38]. The adaptation protocol included interviewing 30 mothers of children from 18 to 30 months of age in Shona-speaking families to identify common words, piloting this list of over 350 words with 30 additional mothers of similar-aged children, identifying those words that correlated with age (*p* > 0.1), and selecting a range of 100 words found to be easy (70%–100% said their child knew the word), moderate (40%–70% said their child knew the word), and hard (only 20%–40% said their child knew the word). This vocabulary checklist was then piloted with another 30 mothers, and inter-rater reliability was tested to ensure 97% agreement between testers on the same list with the same mother [38]. This process was conducted only in Shona-speaking households. We included the CDI grammar checklist for 2-year-olds to increase the number of items specifically targeting language development, which changes dramatically at this age.

The A-not-B test assesses object permanence and working memory [39]. This task requires the child to watch as a treat is hidden under 1 of 2 bowls (A or B); after a brief delay, the child is asked to find the treat under one of the bowls and in doing so, to remember (through object permanence) which one of the bowls it was hidden under (A or B). The exercise is repeated 10 times, switching which bowl the treat is hidden under according to a strict protocol for all 10 episodes to check that the child has no perseveration error. Children not completing a full set of 10 tests were excluded from analysis.

The self-control task [40] we used assesses impulsivity. The child is required to watch as a treat is promised to them, but they have to wait for 2 minutes to take it. The test is first conducted with a covered treat, then with an uncovered treat. Self-control was defined as a child who waited for 2 minutes. We conducted and scored the test in a similar way to that done in Uganda [41].

#### Study outcomes

We prespecified that the primary inferences would be based on children of mothers testing HIV-negative during pregnancy; these results are presented in this paper. Results among children born to HIV-positive mothers will be reported separately.

The prespecified primary outcomes of the ECD substudy were total MDAT score (out of 138), MDAT gross motor score (out of 36), MDAT fine motor score (out of 36), MDAT social score (out of 30), MDAT language score (out of 36), MacArthur–Bates CDI vocabulary checklist (total number of words known out of 100), A-not-B score (out of 10), and the proportion of children with self-control. The prespecified secondary outcome was the proportion of children who used imperatives or the progressive tense, plurals, or combined 2 words as assessed using the MacArthur–Bates CDI grammar checklist.

#### Validation and quality control

We undertook several validation and quality control procedures. Nurses underwent 6-monthly refresher training and standardization (using non-SHINE children), undertaking an ECD assessment that was observed and double-marked by a gold-standard assessor; percentage agreement had to be >85% to pass the standardization. At each standardization (3 in total), nurses were asked to measure 1 child twice (once in the morning and once in the afternoon), for which intra-class correlations for each test were as follows: MDAT, 0.88 (95% CI 0.82, 0.94); MacArthur–Bates CDI, 0.94 (95% CI 0.90, 0.96); A-not-B test, 0.85 (95% CI 0.80, 0.90); and self-control task, 0.80 (95% CI 0.76, 0.85). Supportive supervision of ECD assessments was undertaken during monthly field visits, with corrective or reinforcing feedback provided to nurses. Finally, 5% of assessments in the field were video-recorded. These assessments were then reviewed and double-marked by a psychologist with expertise in all tests (JC) and a pediatrician with advanced training in child neurodevelopment and Shona language proficiency (GK). Percentage agreement was 93% for MDAT fine motor, 90% for MDAT language, 97% for A-not-B, and 91% for the self-control task. Only the nurse’s score was used in the final analysis.

### Statistical analysis

All analyses were intention-to-treat at the child level. For tests with continuous outcomes (MDAT, MacArthur–Bates CDI, and A-not-B test), the absolute difference in mean score between treatment groups was estimated. For tests with dichotomous outcomes (self-control and grammar), the relative risk (RR) of passing the test was estimated in comparing treatment groups. Although the study was not powered to detect a statistical interaction between the IYCF and WASH treatments, it was estimated for each outcome. We accounted for the interaction in the model if it was significant (*p <* 0.05, according to the Wald test) or had a sizeable point estimate (i.e., difference in mean score > 0.25 SD for continuous outcomes; RR > 2 or <0.5 for dichotomous outcomes). Otherwise, we used a regression model with 2 terms to represent the treatment arms; we estimated the effect of IYCF by comparing the 2 IYCF arms with the 2 non-IYCF arms and estimated the effect of WASH by comparing the 2 WASH arms with the 2 non-WASH arms. If interaction was significant, we used a regression model with 3 terms to represent the 4 treatment arms. We used generalized estimating equations that accounted for within-cluster correlation to estimate effect size, unadjusted for other covariates, with an exchangeable working correlation structure [39]. A log-binomial specification was used to facilitate estimation of RRs. We compared baseline characteristics between arms while handling within-cluster correlation using multinomial and ordinal regression models with robust variance estimation, and Somers’ D for medians. We used Stata (version 14.1) for all analyses.

Adjusted analyses controlled for prespecified baseline covariates (as in our statistical analysis plan), which were initially assessed in bivariate analyses to identify those with an important association with the outcome (for dichotomous outcomes: *p <* 0.2 or RR > 2.0 or < 0.5; for continuous outcomes: *p <* 0.2 or difference > 0.25 SD). Selected covariates were entered in a multivariable regression model; a forward stepwise selection procedure was implemented, with *p <* 0.2 required for a variable to enter the model.

A per-protocol analysis was conducted to examine intervention effects when delivered at high fidelity (prespecified for WASH + IYCF arm as receiving all 10 core modules; for other arms predefined as receiving all modules scheduled at the same time-points when WASH + IYCF core modules were delivered). A prespecified subgroup analysis by child sex was planned if the interaction terms were *p <* 0.05. In a prespecified sensitivity analysis, children of women who seroconverted to HIV after pregnancy were excluded.

### Sample size

Other ECD intervention trials and comparisons of preterm versus term children have reported effect sizes of 0.3–0.4 standard deviations for similar ECD outcomes [42,43]. Accordingly, we calculated our sample size requirements to detect a 0.2 standard deviation shift with >80% power and a type I error rate of 5%, assuming an ICC of 0.07, 10 children per cluster, 33 clusters per arm, and a total of 132 clusters. We therefore aimed to recruit at least 1,320 children.

### Trial oversight and registration

The Medical Research Council of Zimbabwe and the Institutional Review Board of the Johns Hopkins Bloomberg School of Public Health approved the study protocol (Zimbabwe: MRCZ/A/1675; Johns Hopkins University: IRB#4205). The ECD substudy protocol was included as an amendment to the main SHINE trial protocol, submitted to and approved by the 2 institutional review boards. The SHINE statistical analysis plan included the prespecified ECD outcomes. These documents can be found in S1 Text and at https://osf.io/w93hy. An independent data and safety monitoring board comprising 2 physicians from Zimbabwe and a statistician from the UK (listed in Acknowledgments) reviewed interim adverse event data in the main trial between enrollment and 18 months of age, but not in the ECD substudy since no interventions were provided between 18 and 24 months of age. The trial was registered at ClinicalTrials.gov (NCT01824940).

## Results

### Enrollment and follow-up

**Fig 1:**
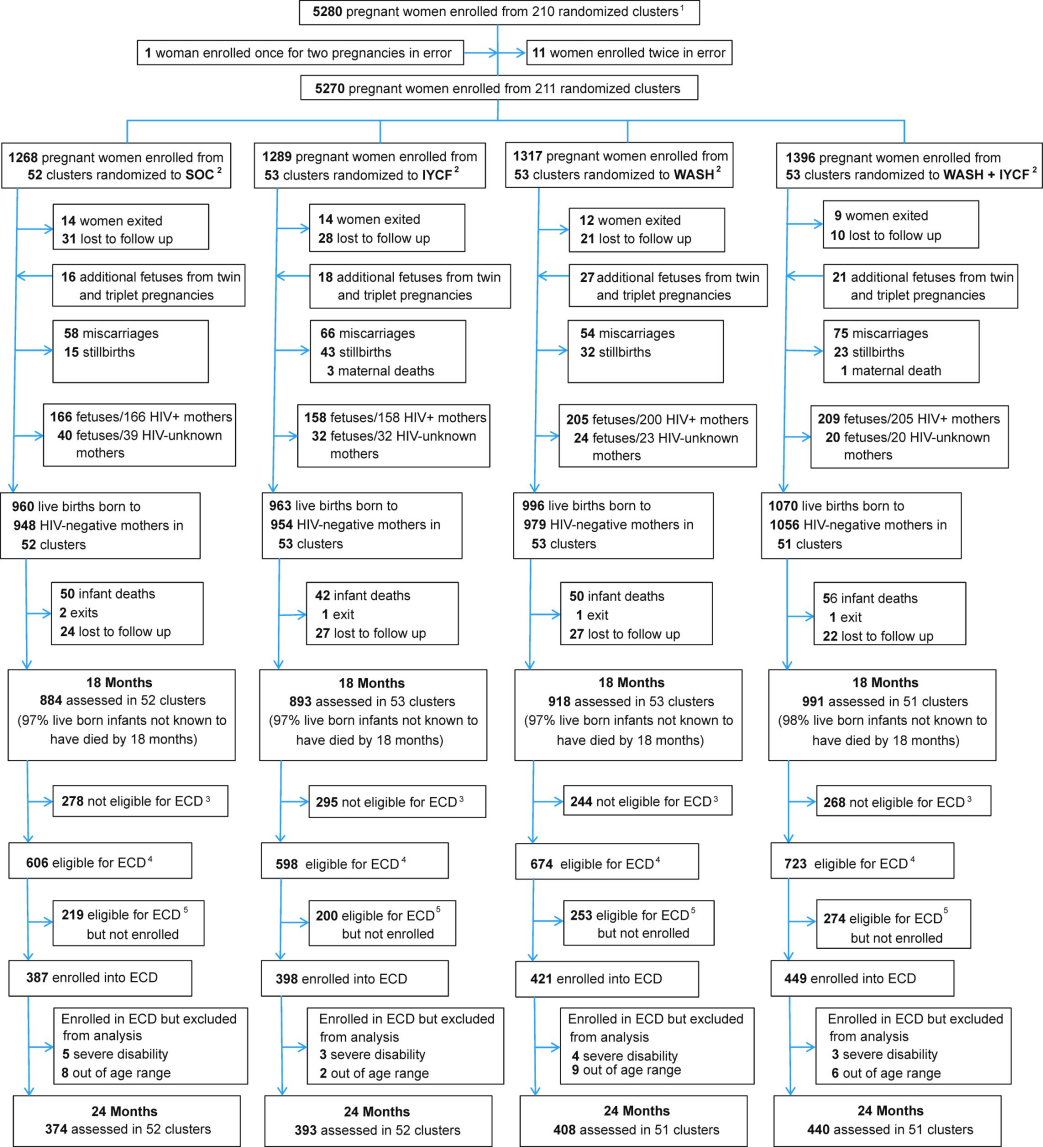
Flow of participants through the SHINE early child development (ECD) substudy. ^1^In all, 212 clusters were randomized, 53 in each of the 4 trial arms. After randomization, 1 cluster was excluded as it was determined to be in an urban area, 1 cluster was excluded as the village health worker covering it mainly had clients outside the study area, and 1 more was merged into a neighboring cluster based on subsequent data on village health worker coverage. Three new cluster designations were created due to anomalies in the original mapping: for 2 of these, the trial arm was clear; the third contained areas that were in 2 trial arms, and was assigned to the underrepresented arm, resulting in 53 clusters in each arm. All of this occurred before enrollment began. When enrollment was completed, however, there was 1 standard-of-care cluster in which no women were enrolled, leaving a total of 211 clusters available for analysis. ^2^SOC, standard of care; IYCF, infant and young child feeding; WASH; water, sanitation, and hygiene.^3^Children were not eligible for the ECD substudy if they turned 2 years of age (allowable range 102–112 weeks) before March 1, 2016.^4^Children were eligible for the ECD substudy if they turned 2 years of age (allowable range 102–112 weeks) between March 1, 2016, and April 30, 2017.^5^Children were eligible for the ECD substudy because they turned 2 years of age (allowable range 102–112 weeks) between March 1, 2016, and April 30, 2017, but they were not contactable or were not approached for consent because the number of children becoming 102–112 weeks of age between March 1, 2016, and April 30, 2017, exceeded the capacity of the 11 ECD-trained nurses.

Between November 22, 2012, and March 27, 2015, 5,280 pregnant women were enrolled from 211 clusters at median 12 (IQR 9, 16) gestational weeks (Fig 1). Of 3,989 HIV-unexposed live births, 198 (4.8%) children died, 5 (0.1%) voluntarily left the study, and 100 (2.5%) were lost to follow-up or moved outside Zimbabwe; 3,686 children were therefore assessed at the 18-month visit. As previously reported, mean length-for-age *Z*-score (LAZ) was 0.16 (95% CI 0.08, 0.23) higher and hemoglobin concentration 2.03 (95% CI 1.28, 2.79) g/l higher among children in the IYCF compared to non-IYCF arms, but there was no evidence that the WASH intervention affected either LAZ or hemoglobin [44]. There was a modest impact on weight by the IYCF intervention, which we have previously reported (increase in weight-for-age *Z*-score: 0.13 [95% CI 0.07, 0.20], *p <* 0.001) [44].

Of the 3,686 children who provided trial primary outcomes at 18 months, 2,601 (70.6%) became 102 weeks to 112 weeks of age during the enrollment period. Of these 2,601 eligible children, 1,655 (63.6%; from 206 clusters) were enrolled in the ECD substudy and assessed at 24 months. The remaining 946 children were not enrolled: 12 (1.3%) declined; 2 (0.2%) died between 18 and 24 months of age; 464 (49.0%) had relocated temporarily or permanently from their study home; 194 (20.5%) could not be scheduled at a mutually agreeable time within the required age window; and 274 (29.0%) were not reachable by telephone or home visit to determine availability and interest in joining the ECD substudy. The mean (SD) age of children at the time of ECD assessment was very similar across trial arms (SOC: 105.3 [2.0] weeks; IYCF: 104.9 [1.9] weeks; WASH: 105.3 [2.0] weeks; WASH + IYCF: 105.2 [2.0] weeks). Fifteen children (0.9%) (5, 3, 4, and 3 from the SOC, IYCF, WASH, and WASH + IYCF arms, respectively) were assessed for ECD but excluded from analysis because they scored “moderate to severe” on the Washington screening tool, and 25 (1.5%) (8, 2, 9, and 6 from the SOC, IYCF, WASH, and WASH + IYCF arms, respectively) were excluded because they were found to be outside the allowable age window of 102–112 weeks, leaving 1,615 HIV-unexposed children in the present analysis. Baseline characteristics of mothers/children who joined and did not join the ECD substudy are shown in Table 1, split into 3 groups: those who were not eligible, those who were eligible but not enrolled, and those who were enrolled.

**Table 1:** Comparison of baseline characteristics across 3 groups of children: children who completed the 18-month visit but were not eligible for the ECD substudy, children who were eligible for but were not enrolled into the ECD substudy, and children who were eligible for and were enrolled into the ECD substudy.

Baseline characteristic^a^	Children who were not eligible for ECD substudy	Children who were eligible but were not enrolled into ECD substudy	Children who were eligible and were enrolled into ECD substudy	*p*-Value
Woman assessed, *N*	1,076	933	1,634	
Children assessed, *N*	1,085	946	1,655	
Women completing baseline visit, *N*	846	846	1,545	
**Household characteristics**				
Size, median (IQR) [*n*]	4 (3,6) [1,064]	5 (3,6) [887]	5 (3,6) [1,554]	0.57
Wealth quintile^b^, percent [*n*]				0.43
Lowest	17.5 [188]	17.4 [162]	16.1 [263]	
Second	17.7 [190]	15.9 [148]	18.8 [307]	
Middle	19.7 [212]	16.8 [157]	19.8 [323]	
Fourth	18.4 [198]	19.0 [177]	20.3 [332]	
Highest	19.2 [207]	20.7 [193]	19.3 [315]	
***Electricity***				
Power grid, percent [*n*]	2.4 [24]	2.9 [24]	3.3 [46]	0.93
Other power, percent [*n*]				0.13
Generator	3.0 [30]	3.6 [30]	3.3 [51]	
Solar	65.9 [656]	70.5 [589]	69.1 [1,062]	
No electricity	31.1 [309]	26.0 [217]	27.6 [425]	
***Sanitation***				
Household members who openly defecate (all)^c^, percent [*n*/*N*]	53.4 [518/971]	48.2 [381/790]	48.4 [704/1,455]	<0.001
Household members who openly defecate (by age group), percent [*n*/*N*]				
0 to <3 years	66.7 [50/75]	62.5 [35/56]	59.6 [56/94]	0.46
3 to <6 years	57.1 [60/105]	57.5 [42/73]	52.0 [80/154]	<0.001
6 to <18 years	49.8 [102/205]	42.2 [73/173]	49.5 [146/295]	<0.001
18 to <70 years	54.1 [252/466]	49.9 [186/373]	47.3 [347/734]	0.056
>70 years	25.0 [3/12]	60.0 [6/10]	50.0 [5/10]	<0.001
Any latrine at household, percent [*n*]	36.1 [357]	37.2 [308]	37.7 [567]	0.73
Improved latrine at household, percent [*n*]	30.3 [300]	32.7 [271]	33.5 [503]	0.30
Improved latrine with well-trodden path, percent [*n*]	26.6 [263]	29.0 [240]	29.6 [445]	0.26
Improved latrine with well-trodden path and not shared, percent [*n*]	23.9 [229]	26.7 [215]	26.8 [388]	0.27
***Water***				
Main source of household drinking water is improved, percent [*n*]	64.5 [645]	63.1 [524]	62.7 [946]	0.74
Treat drinking water to make it safer, percent [*n*]	11.8 [116]	11.8 [97]	13.8 [205]	0.26
One-way walk time to fetch water (min), median (IQR) [*n*]	10 (5, 20) [997]	10 (5, 20) [828]	10 (5, 15) [1,504]	<0.001
Per capita water volume collected in past 24 h (l), mean (SD) [*n*]	9.7 (8.6) [857]	9.8 (11.0) [681]	9.6 (9.4) [1,257]	<0.001
***Hygiene***				
Handwashing station at household, percent [*n*]	5.6 [52]	11.5 [92]	9.4 [135]	<0.001
Handwashing station with water, percent [*n*]	2.4 [22]	4.3 [34]	3.0 [43]	0.04
Handwashing station with water and rubbing agent, percent [*n*]	0.7 [6]	0.5 [4]	1.0 [15]	0.27
Improved floor^d^, percent [*n*]	56.0 [551]	55.9 [463]	54.8 [832]	0.80
Number of chickens, median (IQR) [*n*]	6 (2, 10) [986]	6 (2, 10) [835]	6 (2, 10) [1,537]	0.53
Livestock observed inside the house, percent [*n*]	35.5 [353]	38.2 [320]	39.6 [608]	<0.001
Feces observed in the yard, percent [*n*]	30.2 [300]	30.1 [251]	33.2 [506]	0.20
***Diet quality and food security***				
Household meets minimum dietary diversity score^e^, percent [*n*]	38.8 [344]	37.5 [277]	41.7 [560]	0.14
Coping Strategies Index^f^, median (IQR) [*n*]	1 (0, 8) [965]	0 (0, 5) [818]	1 (0, 7) [1,506]	<0.001
**Maternal characteristics**				
Age (y), mean (SD) [*n*]	26.1 (6.5) [994]	24.1 (6.3) [798]	26.5 (7.7) [1,459]	<0.001
Height (cm), mean (SD) [*n*]	160.2 (5.9) [1,048]	159.8 (5.4) [907]	160.3 (6) [1,590]	<0.001
MUAC (cm), mean (SD) [*n*]	26.7 (3.2) [1,054]	26.0 (3.1) [913]	26.5 (3.2) [1,620]	<0.001
Completed schooling (y), mean (SD) [*n*]	9.6 (2.0) [1,039]	9.6 (1.9) [879]	9.6 (2.0) [1,544]	0.088
Parity, median (IQR) [*n*]	1 (0, 2) [588]	1 (0, 2) [588]	2 (1, 3) [1,189]	<0.001
Married, percent [*n*]	97.1 [1,002]	92.8 [809]	95.8 [1,470]	<0.001
Employed, percent [*n*]	7.6 [76]	8.2 [69]	9.4 [144]	<0.001
Religion, percent [*n*]				0.089
Apostolic	47.0 [463]	48.3 [398]	51.3 [759]	
Other Christian (Pentecostal, Catholic, other Christian)	48.8 [481]	48.9 [403]	46.2 [684]	
Other religion (Muslim and other)	4.2 [41]	2.8 [23]	2.6 [38]	
Maternal capabilities^g^				
Gender norms and attitudes, mean (SD) [*n*]	1.95 (0.89) [984]	2.35 (0.83) [834]	2.25 (1.00) [1,530]	<0.001
Perceived social support, mean (SD) [*n*]	3.5 (0.71) [965]	3.62 (0.76) [813]	3.61 (0.67) [1,503]	<0.001
Perceived physical health, mean (SD) [*n*]	3.35 (1.01) [846]	3.46 (1.09) [723]	3.41 (0.96) [1,331]	<0.001
Mothering self-efficacy, mean (SD) [*n*]	3.97 (0.42) [966]	3.92 (0.43) [814]	3.98 (0.40) [1,513]	<0.001
Perceived time stress, mean (SD) [*n*]	2.71 (0.73) [965]	2.58 (0.80) [818]	2.66 (0.86) [1,507]	<0.001
Decision-making autonomy, median (IQR) [*n*]	5 (3, 5) [910]	5 (4, 5) [760]	5 (4, 5) [1,379]	0.003
**Infant characteristics**				
Female, percent [*n*]	48.6 [527]	50.7 [480]	50.3 [833]	0.51
Birth weight (kg), mean (SD) [*n*]	3.16 (0.44) [927]	3.07 (0.47) [885]	3.11 (0.51) [1,573]	<0.001
Birth weight < 2,500 g, percent [*n*]	5.4 [50]	10.9 [96]	8.2 [129]	<0.001
Institutional delivery, percent [*n*]	89.0 [851]	90.2 [795]	89.3 [138]	0.66
Vaginal delivery, percent [*n*]	92.9 [897]	92.5 [822]	93.1 [1,475]	0.47

^a^Baseline for mothers was 2 weeks after consent (approximately 14 weeks’ gestation). Baseline for infants was at birth.

^b^Per [33].

^c^Open defecation among all household members.

^d^Improved floor defined as concrete, brick, cement, or tile. Unimproved floor defined as mud, earth, sand, or dung.

^e^Per [45].

^f^Coping Strategies Index is a measure of household food insecurity, as described in [46].

^g^Per [47].

MUAC, mid-upper arm circumference.

### Baseline characteristics

Baseline characteristics of enrolled mothers and infants were broadly similar between randomized groups, although there were minor imbalances in wealth, electricity supply, improved water source, water treatment, availability of a handwashing station, observed feces in the yard, and dietary diversity score (Table 2). Almost half of households practiced open defecation, and only one-third had an improved latrine at baseline. Few households had a handwashing station or treated their drinking water. The median walk time to an improved water source was 10 minutes; per capita volume of water collected per day was around 10 liters. Mothers were generally married and well-educated, but very few were employed. Average infant birth weight was 3.10 kg; the majority of infants were born in institutions by normal vaginal delivery.

**Table 2:** Baseline characteristics of mothers and children in the early child development substudy.

Baseline characteristic^a^	SOC^b^	IYCF^b^	WASH^b^	WASH + IYCF^b^
Woman assessed, *N*	383	397	415	444
Children assessed, *N*	387	398	421	449
Women completing baseline visit, *N*	349	368	402	431
**Household characteristics**				
Size, median (IQR) [*n*]	5 (3,6) [363]	5 (3,6) [379]	4 (3,6) [398]	5 (4,6) [419]
Wealth quintile^c^, percent [*n*]				
Lowest	17.2 [66]	13.1 [52]	17.8 [74]	16.2 [72]
Second	18.3 [70]	15.9 [63]	21.0 [87]	19.8 [88]
Middle	18.0 [69]	20.4 [81]	20.5 [85]	20.3 [90]
Fourth	19.3 [74]	20.4 [81]	20.0 [83]	21.4 [95]
Highest	18.0 [69]	22.4 [89]	17.4 [72]	19.1 [85]
***Electricity***				
Power grid, percent [*n*]	3.2 [11]	5.0 [18]	2.7 [11]	1.4 [6]
Other power, percent [*n*]:				
Generator	2.3 [9]	3.6 [14]	4.2 [17]	3.0 [13]
Solar	68.4 [262]	70.3 [279]	68.7 [285]	68.8 [305]
No electricity	29.3 [112]	26.1 [104]	26.1 [108]	28.2 [125]
***Sanitation***				
Household members who openly defecate (all)^d^, percent [*n*/*N*]	49.0 [768/1,566]	49.7 [870/1,752]	48.3 [874/1,809]	45.3 [893/1,972]
Household members who openly defecate (by age group), percent [*n*/*N*]				
0 to <3 years	54.8 [57/104]	60.5 [72/119]	58.5 [55/94]	51.1 [68/133]
3 to <6 years	67.6 [100/148]	56.5 [91/161]	51.2 [106/207]	50.0 [105/210]
6 to <18 years	51.5 [205/398]	50.4 [205/407]	50.1 [208/415]	48.8 [238/488]
18 to <70 years	45.6 [319/700]	49.8 [379/761]	46.2 [377/818]	44.6 [391/877]
>70 years	36.8 [7/19]	33.3 [7/21]	35.7 [5/14]	34.8 [8/23]
Any latrine at household, percent [*n*]	34.3 [118]	39.7 [143]	38.3 [148]	38.0 [159]
Improved latrine at household, percent [*n*]	30.5 [105]	35.6 [128]	34.4 [132]	33.2 [139]
Improved latrine with well-trodden path, percent [*n*]	25.9 [89]	31.9 [115]	30.0 [115]	30.3 [127]
Improved latrine with well-trodden path and not shared, percent [*n*]	24.3 [82]	27.9 [95]	26.9 [99]	27.6 [112]
***Water***				
Main source of household drinking water is improved, percent [*n*]	64.1 [220]	63.4 [229]	58.7 [229]	64.9 [272]
Treat drinking water to make it safer, percent [*n*]	17.8 [60]	15.2 [53]	11.7 [45]	11.5 [48]
One-way walk time to fetch water (min), median (IQR) [*n*]	10 (5, 15) [343]	7 (4, 15) [358]	10 (5, 20) [389]	10 (5, 15) [419]
Per capita water volume collected past 24 h (l), mean (SD) [*n*]	9.5 (9.5) [287]	9.9 (8.9) [298]	9.8 (11.8) [333]	9.1 (7.1) [343]
***Hygiene***				
Handwashing station at household, percent [*n*]	5.0 [16]	2.9 [10]	13.5 [52]	14.4 [57]
Handwashing station with water, percent [*n*]	3.4 [11]	0.6 [2]	4.4 [17]	3.3 [13]
Handwashing station with water and rubbing agent, percent [*n*]	2.2 [7]	0.0 [0]	0.8 [3]	1.3 [5]
Improved floor^e^, percent [*n*]	54.5 [188]	52.8 [190]	54.8 [216]	56.6 [240]
Number of chickens, median (IQR) [*n*]	5 (2, 10) [347]	7 (3, 12) [366]	5 (2, 10) [398]	5 (2, 10) [431]
Livestock observed inside the house, percent [*n*]	38.8 [135]	43.4 [159]	38.8 [155]	37.5 [160]
Feces observed in the yard, percent [*n*]	32.5 [112]	44.1 [160]	31.7 [126]	26.0 [110]
***Diet quality and food security***				
Household meets minimum dietary diversity score^f^	39.9 [122]	46.3 [144]	39.6 [141]	40.8 [153]
Coping Strategies Index^g^_,_ median (IQR) [*n*]	1 (0, 8) [344]	0 (0, 7) [353]	1 (0, 9) [395]	1 (0, 6) [418]
**Maternal characteristics**				
Age (y), mean (SD) [*n*]	26.2 (8.5) [329]	26.0 (7.4) [350]	26.9 (8.1) [382]	26.8 (7.2) [403]
Height (cm), mean (SD) [*n*]	160.3 (5.2) [374]	160.7 (6.4) [386]	160.0 (6.0) [404]	160.3 (5.7) [431]
MUAC (cm), mean (SD) [*n*]	26.4 (3.0) [379]	26.4 (2.6) [391]	26.6 (3.9) [414]	26.5 (2.8) [441]
Completed schooling (y), mean (SD) [*n*]	9.7 (1.8) [363]	9.7 (2.3) [376]	9.5 (1.6) [394]	9.6 (2.1) [416]
Parity, median (IQR) [*n*]	2 (1, 3) [272]	2 (1, 3) [293]	2 (1, 3) [297]	2 (1, 3) [332]
Married, percent [*n*]	95.5 [343]	94.9 [354]	95.7 [376]	96.9 [402]
Employed, percent [*n*]	7.8 [27]	11.3 [41]	9.7 [39]	8.9 [38]
Religion, percent [*n*]				
Apostolic	53.8 [206]	47.5 [189]	51.6 [214]	52.0 [231]
Other Christian (Pentecostal, Catholic, other Christian)	44.8 [172]	51.4 [204]	43.6 [181]	45.2 [201]
Other religion (Muslim and other)	1.4 [5]	1.1 [4]	4.8 [20]	2.8 [12]
Maternal capabilities^h^				
Gender norms and attitudes, mean (SD) [*n*]	2.31 (1.12) [342]	2.34 (0.94) [364]	2.26 (0.94) [402]	2.12 (0.94) [427]
Perceived social support, mean (SD) [*n*]	3.64 (0.57) [338]	3.60 (0.66) [358]	3.59 (0.66) [392]	3.61 (.68) [420]
Perceived physical health, mean (SD) [*n*]	3.43 (0.89) [293]	3.43 (0.77) [323]	3.40 (1.18) [358]	3.39 (1.02) [362]
Mothering self-efficacy, mean (SD) [*n*]	4.00 (0.36) [344]	3.95 (0.40) [357]	3.99 (0.41) [395]	3.98 (0.41) [422]
Perceived time stress, mean (SD) [*n*]	2.63 (1.01) [338]	2.63 (0.66) [358]	2.67 (0.88) [393]	2.70 (0.87) [423]
Decision-making autonomy, median (IQR) [*n*]	5 (4, 5) [319]	5 (4, 5) [327]	5 (3, 5) [350]	5 (3, 5) [387]
**Infant characteristics**				
Female, percent [*n*]	54.0 [209]	49.5 [197]	48.2 [203]	49.9 [224]
Birth weight (kg), mean (SD) [*n*]	3.08 (0.61) [366]	3.08 (0.39) [381]	3.14 (0.52) [399]	3.11 (0.43) [427]
Birth weight < 2,500 g, percent [*n*]	10.7 [41]	6.8 [25]	8.5 [34]	7.3 [31]
Institutional delivery, percent [*n*]	88.8 [325]	88.7 [331]	89.5 [349]	90.1 [381]
Vaginal delivery, percent [*n*]	92.5 [345]	93.7 [358]	93.1 [375]	92.8 [401]

^a^Baseline for mothers was 2 weeks after consent (approximately 14 weeks’ gestation). Baseline for infants was at birth.

^b^SOC, standard of care; IYCF, infant and young child feeding; WASH, water, sanitation, and hygiene.

^c^Per [33].

^d^Open defecation among all household members.

^e^Improved floor defined as concrete, brick, cement, or tile. Unimproved floor defined as mud, earth, sand, or dung.

^f^Per [45].

^g^Coping Strategies Index is a measure of household food insecurity, as described in [46].

^h^Per [47].

MUAC, mid-upper arm circumference.

### Intervention delivery and uptake

Fidelity of intervention implementation was high (Table 3). Among households in the WASH arms, ≥98% received ventilated improved pit latrines, handwashing stations, baby mats, and play yards, and around 90% received ≥80% of planned soap and chlorine solution deliveries. Among IYCF households, almost 90% received ≥80% of planned SQ-LNS deliveries. Across all arms, VHWs completed 90%–95% of planned intervention visits.

**Table 3:** Intervention delivery and participant uptake by treatment group^a^.

Intervention delivery or uptake measure	Data source	Trial arm				WASH			IYCF		
		SOC^b^	IYCF^b^	WASH^b^	WASH + IYCF^b^	Combined WASH^c^	Non-WASH^c^	*p-*Value^d^	Combined IYCF^c^	Non-IYCF^c^	*p-*Value^d^
**Delivery of hardware, supplies, and behavior change modules**											
Number of children with 24-month outcomes on whom inferences are based	Trial logs	374	393	408	440	848	767	—	833	782	—
***WASH supplies***											
SHINE-installed ventilated improved pit latrine	Trial logs	n/a	n/a	99.0	98.6	98.8	n/a	—	n/a	n/a	—
2 handwashing stations (Tippy Taps) delivered	Trial logs	n/a	n/a	100.0	99.8	99.9	n/a	—	n/a	n/a	—
Baby mat delivered	Trial logs	n/a	n/a	99.0	98.9	98.9	n/a	—	n/a	n/a	—
Play yard delivered	Trial logs	n/a	n/a	98.0	98.6	98.4	n/a	—	n/a	n/a	—
Received ≥16 (80% of expected) soap deliveries	Trial logs	n/a	n/a	91.2	90.5	90.8	n/a	—	n/a	n/a	—
Received ≥12 (80% of expected) WaterGuard deliveries	Trial logs	n/a	n/a	91.2	89.8	90.5	n/a	—	n/a	n/a	—
***IYCF supplies***											
Received ≥11 (80% of expected) SQ-LNS deliveries	Trial logs	n/a	88.3	n/a	89.3	n/a	n/a	—	88.8	n/a	—
***Behavior change modules***											
Percent intervention modules completed (percent due)	VHW report	93.2	92.6	95.0	95.0	95.0	92.9	0.017	93.9	94.3	0.69
**Participant behaviors at 12-month visit**											
Number of mothers with 12- and 24-month outcomes	Trial logs	337	359	368	395	763	696	—	754	705	—
Number of children with 12- and 24-month outcomes	Trial logs	340	360	374	399	773	700	—	759	714	—
***WASH-related behaviors***								—			
Household members who practice open defecation	Maternal report	43.4	37.9	0.2	0.9	0.6	40.4	<0.001	n/a	n/a	—
Any latrine at household	Observed	34.5	43.9	100.0	99.8	99.9	39.3	<0.001	n/a	n/a	—
Improved latrine at household	Observed	30.0	36.8	100.0	99.2	99.6	33.5	<0.001	n/a	n/a	—
Improved latrine at household with well-trodden path, not used for storage, and not shared with other households	Observed and maternal report	23.2	26.8	86.9	87.5	87.5	25.0	<0.001	n/a	n/a	—
Handwashing station at household	Observed	5.3	7.9	98.9	97.7	97.7	6.6	<0.001	n/a	n/a	—
Handwashing station with water and rubbing agent at household	Observed	2.5	2.7	85.9	85.3	85.6	2.6	<0.001	n/a	n/a	—
Ever treats drinking water to make it safer	Maternal report	12.8	12.8	89.7	87.0	88.3	12.8	<0.001	n/a	n/a	—
Disposes rinse water from cleaning infant nappies with feces in a latrine	Maternal report	32.5	36.8	77.2	79.3	78.3	34.7	<0.001	n/a	n/a	—
Play yard is visibly clean	Observed	n/a	n/a	92.3	92.3	92.3	n/a	n/a	n/a	n/a	—
Child ever observed to eat soil	Maternal report	78.7	68.6	24.3	28.3	26.4	73.5	<0.001	n/a	n/a	—
Child ever observed to eat chicken feces	Maternal report	24.9	17.7	2.7	3.1	2.9	21.2	<0.001	n/a	n/a	—
***IYCF behaviors***											
Child is still breastfeeding	Maternal report	97.3	97.5	97.6	96.5	n/a	n/a	—	96.9	97.5	0.55
Mother reports correct ways to feed child during and after illness	Maternal report	61.6	66.7	62.2	69.7	n/a	n/a	—	68.3	61.9	0.017
Infant diet met minimum dietary diversity in past 24 hours^e^	Maternal report	52.6	70.4	55.4	71.1	n/a	n/a	—	70.8	54.0	<0.001
Infant consumed iron-rich food in the past 24 hours	Maternal report	50.0	96.9	48.9	95.7	n/a	n/a	—	96.3	49.4	<0.001
Infant consumed animal-source food in the past 24 hours	Maternal report	62.0	70.4	61.7	70.3	n/a	n/a	—	70.3	61.9	0.003
Infant consumed vitamin-A-rich food in the past 24 hours	Maternal report	68.5	80.6	66.9	78.1	n/a	n/a	—	79.3	67.6	<0.001
SQ-LNS consumed in previous 24 hours	Maternal report	n/a	96.2	n/a	90.5	n/a	n/a	—	93.2	n/a	n/a

^a^Data are percent, unless otherwise indicated.

^b^SOC, standard of care; IYCF, infant and young child feeding; WASH, water, sanitation, and hygiene; WASH + IYCF, both IYCF and WASH implemented together.

^c^Combined WASH collapses the 2 WASH-containing arms (WASH and WASH + IYCF); non-WASH collapses the 2 arms not including WASH (SOC and IYCF). Combined IYCF collapses the 2 IYCF-containing arms (IYCF and WASH + IYCF); non-IYCF collapses the 2 arms not including IYCF (SOC and WASH).

^d^*p*-Values adjusted for clustering effect. Depending on the variable type, generalized estimating equation, multinomial, or ordinal regression models with robust variance estimation, or Somers’ D for medians, were used for comparing arms while handling within-cluster correlation.

^e^Per [45].

n/a, not applicable; SQ-LNS, small-quantity lipid-based nutrient supplement; VHW, village health worker.

Intervention implementation, assessed by observed and reported behaviors at the 12-month postnatal visit, achieved substantial contrast between arms (Table 3). In the WASH arms, open defecation among household members was virtually eliminated (0.6% compared to 40.4% in the non-WASH arms). Almost all households in the WASH arms (>99%) had an improved latrine; in 87% of households, the latrine had a well-trodden path and was not being used for storage (compared to 25% in non-WASH arms). In all, 85.6% of WASH households had a handwashing station with observed soap or rubbing agent and water (compared to 2.6% of non-WASH households). Among WASH households compared to non-WASH households, 26.4% versus 73.5% of mothers reported ever seeing their child ingest soil, and 2.9% versus 21.2% reported ever seeing their child ingest chicken feces. Compared to children in the non-IYCF arms, a higher proportion of children in the IYCF arms met minimum dietary diversity, and children in the IYCF arms had consumed more animal-source, more iron-rich, and more vitamin-A-rich foods in the previous day; >90% of children in the IYCF arms consumed SQ-LNS in the previous day. More than 95% of infants in all groups were still being breastfed at 12 months.

### Primary outcomes

The effects of the randomized interventions on primary ECD outcomes at 24 months are shown in Table 4. For all ECD outcomes presented in this paper, there was no interaction between the IYCF and WASH treatments; accordingly, we estimated the effects of the interventions by comparing the 2 IYCF groups with the 2 non-IYCF groups and the 2 WASH groups with the 2 non-WASH groups. The IYCF intervention had a small but significant effect on the total MDAT score (unadjusted difference 1.35, 95% CI 0.24, 2.46; *p =* 0.02), which was non-significant on adjustment (adjusted difference 0.79, 95% CI −0.22, 1.60; *p =* 0.06). This effect size corresponds to a 0.15-SD increase in total MDAT score among children randomized to the IYCF intervention. The total MDAT score difference was driven by slightly higher scores in the language component (unadjusted difference 0.66, 95% CI 0.12, 1.19; *p =* 0.02) and social component (unadjusted difference 0.26, 95% CI 0.01, 0.51; *p =* 0.04) for children in the IYCF groups; both differences were attenuated in adjusted analyses (Table 4). We found no evidence that the WASH intervention affected the total MDAT score or any of its components.

**Table 4:** Effect of WASH and IYCF interventions on early child development at 24 months of age.

Outcome	Effects by arm			Main effects combining arms							
	Treatment group	*N*	Mean (SD) or percent	Treatment group		*N*	Mean (SD) or percent	Unadjusted difference in mean score (95% CI)	*p-*Value	Adjusted^a^ difference in mean score (95% CI)	*p-*Value
**Primary continuous outcomes, mean (SD)**											
MDAT total score^b^	SOC	374	92.7 (9.5)	IYCF: no		782	92.2 (9.4)	0.0 (ref)		0.0 (ref)	
	IYCF	393	93.0 (8.7)	IYCF: yes		833	93.4 (8.9)	1.35 (0.24, 2.46)	0.017	0.79 (−0.22, 1.60)	0.057
	WASH	408	91.7 (9.2)	WASH: no		767	92.8 (9.1)	0.0 (ref)		0.0 (ref)	
	WASH + IYCF	440	93.8 (9.1)	WASH: yes		848	92.8 (9.3)	0.04 (−1.07, 1.15)	0.94	0.32 (−0.51, 1.16)	0.45
MDAT gross motor	SOC	374	23.8 (3.3)	IYCF: no		782	23.6 (3.2)	0.0 (ref)		0.0 (ref)	
	IYCF	393	23.8 (3.1)	IYCF: yes		833	23.9 (3.2)	0.27 (−0.10, 0.64)	0.15	0.08 (−0.17, 0.34)	0.51
	WASH	408	23.4 (3.0)	WASH: no		767	23.8 (3.2)	0.0 (ref)		0.0 (ref)	
	WASH + IYCF	440	23.9 (3.4)	WASH: yes		848	23.7 (3.2)	−0.13 (0.50, 0.24)	0.51	−0.01 (−0.28, 0.26)	0.94
MDAT fine motor	SOC	374	23.4 (2.7)	IYCF: no		782	23.2 (2.5)	0.0 (ref)		0.0 (ref)	
	IYCF	393	23.2 (2.5)	IYCF: yes		833	23.4 (2.4)	0.17 (−0.10, 0.45)	0.21	0.15 (−0.08, 0.37)	0.22
	WASH	408	23.1 (2.3)	WASH: no		767	23.3 (2.6)	0.0 (ref)		0.0 (ref)	
	WASH + IYCF	440	23.6 (2.2)	WASH: yes		848	23.4 (2.3)	0.10 (−0.18, 0.37)	0.49	0.17 (−0.07, 0.41)	0.17
MDAT language	SOC	374	21.4 (4.2)	IYCF: no		782	21.2 (4.2)	0.0 (ref)		0.0 (ref)	
	IYCF	393	21.7 (4.2)	IYCF: yes		833	21.8 (4.2)	0.66 (0.12, 1.19)	0.016	0.33 (−0.07, 0.74)	0.11
	WASH	408	21.0 (4.2)	WASH: no		767	21.5 (4.2)	0.0 (ref)		0.0 (ref)	
	WASH + IYCF	440	21.9 (4.2)	WASH: yes		848	21.5 (4.2)	−0.06 (−0.60, 0.48)	0.83	0.04 (−0.38, 0.46)	0.85
MDAT social	SOC	374	24.2 (2.1)	IYCF: no		782	24.1 (2.4)	0.0 (ref)		0.0 (ref)	
	IYCF	393	24.3 (2.1)	IYCF: yes		833	24.4 (2.1)	0.26 (0.01, 0.51)	0.038	0.22 (0.03, 0.41)	0.025
	WASH	408	24.1 (2.7)	WASH: no		767	24.2 (2.1)	0.0 (ref)		0.0 (ref)	
	WASH + IYCF	440	24.5 (2.2)	WASH: yes		848	24.3 (2.5)	0.10 (−0.14, 0.35)	0.41	0.13 (−0.07, 0.34)	0.19
McArthur–Bates CDI^c^	SOC	366	61.3 (18.7)	IYCF: no		765	61.3 (18.9)	0.0 (ref)		0.0 (ref)	
	IYCF	379	61.3 (18.7)	IYCF: yes		809	61.5 (18.8)	1.65 (−0.52, 3.81)	0.14	0.81 (−1.07, 2.68)	0.40
	WASH	399	61.2 (19.1)	WASH: no		745	61.3 (18.7)	0.0 (ref)		0.0 (ref)	
	WASH + IYCF	430	63.6 (18.8)	WASH: yes		829	62.4 (19.0)	0.99 (−1.18, 3.17)	0.37	1.81 (0.01, 3.61)	0.049
A-not-B test^d^	SOC	352	7.8 (1.3)	IYCF: no		726	7.8 (1.3)	0.0 (ref)		0.0 (ref)	
	IYCF	368	7.7 (1.4)	IYCF: yes		779	7.8 (1.4)	0.05 (−0.11, 0.21)	0.57	0.04 (−0.11, 0.19)	0.62
	WASH	374	7.7 (1.4)	WASH: no		720	7.8 (1.4)	0.0 (ref)		0.0 (ref)	
	WASH + IYCF	411	7.9 (1.4)	WASH: yes		785	7.8 (1.4)	−0.01 (−0.17, 0.15)	0.90	−0.01 (−0.16, 0.15)	0.94
**Primary dichotomous outcomes, percent of children with self-control**^e^											
Self-control task (hidden)^f^	SOC	366	64.8	IYCF: no		764	64.0	1.00 (ref)		1.00 (ref)	
	IYCF	387	64.1	IYCF: yes		826	64.6	0.98 (0.86, 1.12)	0.78	0.93 (0.82, 1.06)	0.28
	WASH	398	63.3	WASH: no		753	64.4	1.00 (ref)		1.00 (ref)	
	WASH + IYCF	439	65.2	WASH: yes		837	64.3	1.01 (0.88, 1.15)	0.94	1.02 (0.90, 1.17)	0.72
Self-control task (unhidden)^g^	SOC	360	47.2	IYCF: no		756	45.4	1.00 (ref)		1.00 (ref)	
	IYCF	385	45.5	IYCF: yes		821	46.2	0.98 (0.90, 1.07)	0.70	0.97 (0.90, 1.05)	0.43
	WASH	396	43.7	WASH: no		745	46.3	1.00 (ref)		1.00 (ref)	
	WASH + IYCF	436	46.8	WASH: yes		832	45.3	1.03 (0.94, 1.12)	0.54	0.99 (0.91, 1.08)	0.86
**Secondary dichotomous language outcomes, percent**											
Uses plurals	SOC	374	16.3	IYCF: no	782		21.0	1.00 (ref)		1.00 (ref)	
	IYCF	393	25.7	IYCF: yes	833		26.3	1.29 (0.96, 1.73)	0.088	1.23 (1.04, 1.45)	0.013
	WASH	408	25.2	WASH: no	767		21.1	1.00 (ref)		1.00 (ref)	
	WASH + IYCF	440	26.8	WASH: yes	848		26.1	1.21 (0.91, 1.61)	0.19	1.30 (1.09, 1.55)	0.003
Combines 2 words	SOC	374	98.9	IYCF: no	782		98.6	1.00 (ref)		1.00 (ref)	
	IYCF	393	99.0	IYCF: yes	833		98.7	1.00 (0.99, 1.01)	0.87	1.00 (0.99, 1.01)	0.87
	WASH	408	98.3	WASH: no	767		99.0	1.00 (ref)		1.00 (ref)	
	WASH + IYCF	440	98.4	WASH: yes	848		98.3	0.99 (0.98, 1.00)	0.26	1.00 (0.99, 1.01)	0.53
Uses imperatives/progressives	SOC	374	76.2	IYCF: no	782		74.8	1.00 (ref)		1.00 (ref)	
	IYCF	393	67.4	IYCF: yes	833		71.5	0.96 (0.90, 1.02)	0.18	0.97 (0.92, 1.02)	0.20
	WASH	408	73.5	WASH: no	767		71.7	1.00 (ref)		1.00 (ref)	
	WASH + IYCF	440	75.2	WASH: yes	848		74.4	1.04 (0.97, 1.11)	0.27	1.04 (0.98, 1.09)	0.20

^a^Adjusted for mother’s mid-upper arm circumference, mother’s education, mother’s employment, maternal capabilities, improved latrine, low birth weight, prematurity, infant sex, calendar month, fieldworker, and decimal age of the child.

^b^Maximum MDAT scores in children up to 5 years: MDAT total, 138; MDAT gross motor, 36; MDAT fine motor, 36; MDAT language, 36; and MDAT social, 30.

^c^In all, 41 participants were removed from the MacArthur–Bates analysis as Shona was not regularly spoken at home to the child (8 from SOC, 14 from IYCF, 9 from WASH, 10 from WASH + IYCF).

^d^In all, 110 participants were removed from the A-not-B test as they did not complete all 10 trials of the test, which was an inclusion criterion for this assessment, as stated in the Methods (22 from SOC, 25 from IYCF, 34 from WASH, 29 from WASH + IYCF).

^e^Defined as children who waited 2 minutes before taking a treat. Reported is relative risk of no self-control (1 − *p*).

^f^In all, 25 participants were removed from the self-control (hidden) test as they had incomplete data (8 from SOC, 6 from IYCF, 10 from WASH, 1 from WASH + IYCF).

^g^In all, 38 participants were removed from the self-control (unhidden) test as they had incomplete data (14 from SOC, 8 from IYCF, 12 from WASH, 4 from WASH + IYCF).

CDI, Communicative Development Inventories; IYCF, infant and young child feeding; MDAT, Malawi Developmental Assessment Tool; SOC, standard of care; WASH, water, sanitation, and hygiene.

There was no impact of IYCF on the MacArthur–Bates CDI grammar checklist in unadjusted or adjusted analyses. The WASH intervention had no impact in unadjusted analyses, but on adjustment it had a small but significant impact on the total number of words reported to be used by the child (adjusted difference 1.81, 95% CI 0.01, 3.61; *p =* 0.049). This effect size corresponds to a 0.09 SD increase in MacArthur–Bates CDI score among children randomized to the WASH intervention.

Neither intervention had any evidence of impact on the A-not-B test or self-control task in unadjusted or adjusted analyses (Table 4).

### Secondary outcomes

The IYCF intervention had a small but significant impact on the proportion of children reported to use plurals (adjusted RR 1.23, 95% CI 1.04, 1.45; *p =* 0.013) but no evidence of impact on the proportion of children reported to combine 2 words or the proportion of children using imperatives or the progressive tense. The WASH intervention had a significant impact on the use of plurals (adjusted RR 1.30, 95% CI 1.09, 1.55; *p =* 0.003) but had no evidence of impact on either the proportion of children reported to combine 2 words or the proportion of children reported to use imperatives or the progressive tense.

### Sensitivity analyses

In the per-protocol analysis, effects of IYCF and WASH among the 1,310 children of mothers who had high-fidelity intervention delivery showed slightly reduced point estimates compared to the intention-to-treat findings, and differences between arms were no longer significant (S1 Table). In a preplanned subgroup analysis, there was no interaction between treatment group and child sex.

## Discussion

We investigated the independent and combined effects of improved WASH and improved IYCF on ECD in a setting of high stunting and poverty in rural Zimbabwe. Overall, we found little evidence that either package of interventions improved child development scores at 2 years of age. There was a small but significant impact of the IYCF intervention on unadjusted MDAT total, language, and social developmental scores; however, the differences between IYCF and non-IYCF groups were extremely modest (<1 item on the MDAT) and not significant in adjusted analyses. There was a small but significant impact of the WASH intervention in adjusted analyses for the CDI language test (which was not present in unadjusted analyses), but this was not reflected in the MDAT language score. There was no impact of WASH on any other ECD test.

Previous studies have reported larger effects on psychomotor development (i.e., changes of 2 to 8 points on the Bayley or Griffiths scales) following an intervention to improve complementary feeding [42,48,49]. In our study the IYCF intervention increased the overall MDAT score by only 1–2 points (0.15 standard deviations) in unadjusted analyses, equivalent to a child completing 1 or 2 extra tasks at the age of 2 years (e.g., running, saying 2 words together, or being able to thread beads or stack objects). These findings are consistent with the main trial results, in which IYCF modestly increased LAZ, head circumference-for-age *Z*-score, and hemoglobin concentration; however, these improvements in growth and anemia appear to translate into very small measurable differences in child development scores. Although the MDAT is a direct assessment tool with good cultural validity, it includes fewer items per age band than the Griffiths assessment (approximately 8 items per age band rather than 12), making it easier to use in a large trial in a rural Zimbabwean setting but potentially less sensitive to change. We did not separate out items in the MDAT to see if children achieved individual items earlier [50] as we felt it was important to concentrate on the prespecified overall global developmental effect; however, this may be an interesting future analysis. Although we found a higher reported number of words spoken by children in the WASH arms, this finding was apparent only in adjusted analyses and the effect size was very small (an additional 1.8 words in the WASH group, equivalent to <0.1 standard deviations of improvement in language).

We have previously highlighted that the interventions typically delivered by WASH programs in rural areas of low- and middle-income countries are insufficiently effective to reduce highly contaminated environments enough to reduce diarrhea or promote linear growth [44]. We have argued that a paradigm shift is needed in the way WASH is delivered, to develop interventions that are more effective and less reliant on behavior change. Whether a more comprehensive and effective WASH intervention can confer benefits for ECD requires evaluation in future studies. Two recent WASH Benefits trials implemented similar interventions to SHINE and evaluated ECD outcomes. In the Kenyan WASH Benefits trial [50], there were no differences in ECD measures at 2 years in either IYCF or WASH intervention groups. By contrast, the Bangladesh WASH Benefits trial [51] found an impact of every WASH intervention delivered singly or in combination, and of the nutrition intervention alone or combined with WASH, on multiple ECD outcomes. However, this trial compared each intervention to a control arm in which families received no promoter visits, making it difficult to disentangle the effects of the interventions from the impact of regular home visits. It is well established that home visiting with promotion of sensitive caregiving can impact ECD [42], and it is plausible that home visiting alone has similar benefits [52].

Our study has strengths and limitations. We undertook one of the only cluster-randomized trials of WASH interventions in early life with and without an IYCF intervention. The current analysis was a substudy of the larger trial, which was primarily designed to evaluate effects of the interventions on linear growth and hemoglobin. We designed the substudy to include a broad assessment of ECD, including executive function, memory, and self-control (often not evaluated at young ages); undertook extensive piloting and validation; and conducted regular quality control checks. Despite this, cognitive tests available to assess 2-year-old children are less sensitive than more complex cognitive assessments used at older ages. Furthermore, although the tests we used have shown sensitivity to change in other field studies [43,53], more sensitive tests or biological techniques such as EEG [54,55] may have given us information that these assessments did not; however, these other approaches are expensive and very difficult to do in field studies. Testing at older ages, such as school entry, would be helpful because tests may be more sensitive to small changes in cognitive function at this age.

We used a constrained randomization technique to mitigate any imbalances during enrollment, and conducted unadjusted and adjusted analyses for all ECD outcomes, using a large number of prespecified covariates to increase the precision of our estimates. In general, the point estimates of the effects of the interventions on ECD outcomes were attenuated after adjustment, although the effect of WASH on the MacArthur–Bates CDI score increased after adjustment. In evaluating the public health impact of the IYCF and WASH interventions, it is important to interpret both models. The adjusted analyses included several trial-related factors (child age, fieldworker, and calendar time) that may have an important influence on ECD measurement; the differences between unadjusted and adjusted estimates may therefore partly reflect the challenge of conducting child development assessments at 2 years of age. Although we adjusted for multiple factors known to influence child development (including socioeconomic status, maternal education, low birth weight, and prematurity), there are likely to be other unmeasured factors influencing child development. The current analysis did not investigate whether or not intermediate factors may have been affected by the WASH intervention, which could interplay with ECD (e.g., mother–child interaction or maternal capabilities); future analyses will study these interactions in more detail.

In summary, we found little effect of improved complementary feeding or an elementary household-level WASH intervention on measures of child development at 2 years of age. There was a very small increase in the total child development (MDAT) score among children receiving the IYCF intervention (in unadjusted analysis only) and a very small increase in language score among children receiving the WASH intervention (in adjusted analysis only); these small effects suggest that neither intervention at scale would meaningfully impact the compromised neurodevelopment that affects 43% of children under 5 years old globally. Neurodevelopment is a complex process impacted by multiple factors, not all of which could be addressed in this trial, such as low birth weight, prematurity, mother–child interaction, poverty, and child stimulation. Collectively, our data suggest that more holistic approaches and interventions that explicitly target ECD, as recommended in the Nurturing Care Framework, may be required to substantially improve child development [52,56].

## Supporting information

S1 Checklist CONSORT checklist. (DOCX)

S1 Protocol Trial protocol. (DOCX)

S1 Table Baseline characteristics of mothers and infants who enrolled and did not enroll in the ECD substudy. (DOCX)

S1 Text Supplementary methods. (DOCX)

S2 Text SHINE Trial Team. (XLSX)

## Abbreviations

CDI: Communicative Development Inventories
ECD: early child development
EED: environmental enteric dysfunction
IYCF: infant and young child feeding
LAZ: length-for-age *Z*-score
MDAT: Malawi Developmental Assessment Tool
MUAC: mid-upper arm circumference
PMTCT: prevention of mother-to-child-transmission
RR: relative risk
SHINE: Sanitation Hygiene Infant Nutrition Efficacy
SOC: standard of care
SQ-LNS: small-quantity lipid-based nutrient supplement
VHW: village health worker
WASH: water, sanitation, and hygiene
