# Supplementary material for: Independent and combined effects of improved water, sanitation, and hygiene (WASH) and improved complementary feeding on early neurodevelopment among children born to HIV-negative mothers in rural Zimbabwe: Substudy of a cluster-randomized trial
Source: PLoS Med. 2019 Mar 21;16(3):e1002766. doi: 10.1371/journal.pmed.1002766 (PMC6428259; doi:10.1371/journal.pmed.1002766)
Supplement: S1 Checklist — (DOCX) [file pmed.1002766.s001.docx]

**Table 1: CONSORT 2010 checklist of information to include when reporting a cluster randomised trial**

| Section/Topic | Item No | Standard Checklist item | Extension for cluster designs | Page No * |
| --- | --- | --- | --- | --- |
| Title and abstract | | | |  |
|  | 1a | Identification as a randomised trial in the title | Identification as a cluster randomised trial in the title | Title |
|  | 1b | Structured summary of trial design, methods, results, and conclusions (for specific guidance see CONSORT for abstracts)^[[1]](#endnote-1),^^[[2]](#endnote-2)^ | See table 2 | Abstract methods |
| Introduction | | | | Introduction |
| Background and objectives | 2a | Scientific background and explanation of rationale | Rationale for using a cluster design | Introduction paragraphs 1-3 |
|  | 2b | Specific objectives or hypotheses | Whether objectives pertain to the the cluster level, the individual participant level or both | Introduction paragraph 4. |
| Methods | | | |  |
| Trial design | 3a | Description of trial design (such as parallel, factorial) including allocation ratio | Definition of cluster and description of how the design features apply to the clusters | Methods for The Shine Trial paragraph 1 and Early Child Development Substudy paragraph 1 and 2 (also see supplementary materials) |
|  | 3b | Important changes to methods after trial commencement (such as eligibility criteria), with reasons |  | N/A |
| Participants | 4a | Eligibility criteria for participants | Eligibility criteria for clusters | Methods/Early Child Development Substudy paragraph 1 and 2 |
|  | 4b | Settings and locations where the data were collected |  | Methods for The Shine Trial paragraph 1 (and supplementary material) |
| Interventions | 5 | The interventions for each group with sufficient details to allow replication, including how and when they were actually administered | Whether interventions pertain to the cluster level, the individual participant level or both | Methods/Intervention Delivery/paragraph 1- 6 (also in supplementary files) |
| Outcomes | 6a | Completely defined pre-specified primary and secondary outcome measures, including how and when they were assessed | Whether outcome measures pertain to the cluster level, the individual participant level or both | Methods/Data Collection/paragraph 1-3 and Methods/ECD Substudy/Assessment Tools paragraph 1-5 and Methods/ECD Substudy/Study outcomes paragraph 1-2 |
|  | 6b | Any changes to trial outcomes after the trial commenced, with reasons |  | none |
| Sample size | 7a | How sample size was determined | Method of calculation, number of clusters(s) (and whether equal or unequal cluster sizes are assumed), cluster size, a coefficient of intracluster correlation (ICC or *k*), and an indication of its uncertainty | Methods/Statistical analysis/sample size paragraph 1 and 2. |
|  | 7b | When applicable, explanation of any interim analyses and stopping guidelines |  | none |
| Randomisation: | | | | Methods/The Shine Trial with details in reference 28 and in supplementary methods section |
| Sequence generation | 8a | Method used to generate the random allocation sequence |  | Methods/The Shine Trial with details in reference 28 and in supplementary methods section |
|  | 8b | Type of randomisation; details of any restriction (such as blocking and block size) | Details of stratification or matching if used | none |
| Allocation concealment mechanism | 9 | Mechanism used to implement the random allocation sequence (such as sequentially numbered containers), describing any steps taken to conceal the sequence until interventions were assigned | Specification that allocation was based on clusters rather than individuals and whether allocation concealment (if any) was at the cluster level, the individual participant level or both | Methods/The Shine Trial with details in reference 28 and in supplementary methods section |
| Implementation | 10 | Who generated the random allocation sequence, who enrolled participants, and who assigned participants to interventions | Replace by 10a, 10b and 10c | Methods/The Shine Trial/Intervention delivery paragraphs 1-6 with details in reference 28 and in supplementary methods section |
|  | 10a |  | Who generated the random allocation sequence, who enrolled clusters, and who assigned clusters to interventions | Methods/The Shine Trial/Intervention delivery paragraphs 1-6 with details in reference 28 and in supplementary methods section |
|  | 10b |  | Mechanism by which individual participants were included in clusters for the purposes of the trial (such as complete enumeration, random sampling) | Methods/The Shine Trial/Intervention delivery paragraphs 1-6 with details in reference 28 and in supplementary methods section VHW workers conducted prospective pregnancy surveillance. |
|  | 10c |  | From whom consent was sought (representatives of the cluster, or individual cluster members, or both), and whether consent was sought before or after randomisation | Women provided written informed consent. Methods/The Shine Trial/Intervention delivery paragraphs 1-6 with details in reference 28 and in supplementary methods section and in supplementary methods section |
|  |  |  |  |  |
| Blinding | 11a | If done, who was blinded after assignment to interventions (for example, participants, care providers, those assessing outcomes) and how |  | Masking of participants and fieldworkers was not possible, but investigators were blinded to trial arm. Methods/The Shine Trial/Intervention delivery paragraphs 1-6 with details in reference 28 and in supplementary methods section |
|  | 11b | If relevant, description of the similarity of interventions |  | Methods/The Shine Trial/Intervention delivery paragraphs 1-6 with details in reference 28. Intervention intensity was the same across arms (all women received same number of Village Health Worker visits and all received the messages delivered in the standard of care (control arm). |
| Statistical methods | 12a | Statistical methods used to compare groups for primary and secondary outcomes | How clustering was taken into account | Methods/Statistical analyses paragraph 1 and 2 |
|  | 12b | Methods for additional analyses, such as subgroup analyses and adjusted analyses |  | Methods/Statistical analyses paragraph 1 and 2 |
| Results | | | |  |
| Participant flow (a diagram is strongly recommended) | 13a | For each group, the numbers of participants who were randomly assigned, received intended treatment, and were analysed for the primary outcome | For each group, the numbers of clusters that were randomly assigned, received intended treatment, and were analysed for the primary outcome | Figure 1 (flow of participants), Table 2 (fidelity and uptake of intervention), narrative in Results/Enrolment and Follow up paragraph 1 and 2. |
|  | 13b | For each group, losses and exclusions after randomisation, together with reasons | For each group, losses and exclusions for both clusters and individual cluster members | Figure 1 (flow of participants), Table 2 (fidelity and uptake of intervention), narrative in supplementary file for main SHINE trial and in Results/Enrolment paragraph 2. |
| Recruitment | 14a | Dates defining the periods of recruitment and follow-up |  | Results/Enrolment and Follow up paragraph 1. |
|  | 14b | Why the trial ended or was stopped |  | Results/Enrolment and Follow up paragraph 1 (Substudy was completed dependent on dates of enrolment into substudy) |
| Baseline data | 15 | A table showing baseline demographic and clinical characteristics for each group | Baseline characteristics for the individual and cluster levels as applicable for each group | Table 1 and Table 2 |
| Numbers analysed | 16 | For each group, number of participants (denominator) included in each analysis and whether the analysis was by original assigned groups | For each group, number of clusters included in each analysis | Table 3 and figure 1 narrative. |
| Outcomes and estimation | 17a | For each primary and secondary outcome, results for each group, and the estimated effect size and its precision (such as 95% confidence interval) | Results at the individual or cluster level as applicable and a coefficient of intracluster correlation (ICC or k) for each primary outcome | Table 3 |
|  | 17b | For binary outcomes, presentation of both absolute and relative effect sizes is recommended |  | Table 3. |
| Ancillary analyses | 18 | Results of any other analyses performed, including subgroup analyses and adjusted analyses, distinguishing pre-specified from exploratory |  | Results/Sensitivity analyses paragraph 1 |
| Harms | 19 | All important harms or unintended effects in each group (for specific guidance see CONSORT for harms^[[3]](#endnote-3)^) |  | Methods for ascertainment in supplmentary methods file. |
| Discussion | | | |  |
| Limitations | 20 | Trial limitations, addressing sources of potential bias, imprecision, and, if relevant, multiplicity of analyses |  | Discussion paragraph 4 |
| Generalisability | 21 | Generalisability (external validity, applicability) of the trial findings | Generalisability to clusters and/or individual participants (as relevant) | Discussion paragraph 6 |
| Interpretation | 22 | Interpretation consistent with results, balancing benefits and harms, and considering other relevant evidence |  | Discussion paragraph 1-6 |
| Other information | | |  |  |
| Registration | 23 | Registration number and name of trial registry |  | Methods/Trial oversight and registration and Abstract last line |
| Protocol | 24 | Where the full trial protocol can be accessed, if available |  | Methods/The Shine Trial and supplementary file 1 and reference 28 |
| Funding | 25 | Sources of funding and other support (such as supply of drugs), role of funders |  | Methods/Trial oversight and registration |

** Note: page numbers optional depending on journal requirements*

**Table 2: Extension of CONSORT for abstracts**1**^,^**2 **to reports of cluster randomised trials**

| Item | Standard Checklist item | Extension for cluster trials |
| --- | --- | --- |
| Title | Identification of study as randomised | Identification of study as cluster randomised |
| Trial design | Description of the trial design (e.g. parallel, cluster, non-inferiority) |  |
| Methods |  |  |
| Participants | Eligibility criteria for participants and the settings where the data were collected | Eligibility criteria for clusters |
| Interventions | Interventions intended for each group |  |
| Objective | Specific objective or hypothesis | Whether objective or hypothesis pertains to the cluster level, the individual participant level or both |
| Outcome | Clearly defined primary outcome for this report | Whether the primary outcome pertains to the cluster level, the individual participant level or both |
| Randomization | How participants were allocated to interventions | How clusters were allocated to interventions |
| Blinding (masking) | Whether or not participants, care givers, and those assessing the outcomes were blinded to group assignment |  |
| Results |  |  |
| Numbers randomized | Number of participants randomized to each group | Number of clusters randomized to each group |
| Recruitment | Trial status^^[[4]](#footnote-1)^^ |  |
| Numbers analysed | Number of participants analysed in each group | Number of clusters analysed in each group |
| Outcome | For the primary outcome, a result for each group and the estimated effect size and its precision | Results at the cluster or individual participant level as applicable for each primary outcome |
| Harms | Important adverse events or side effects |  |
| Conclusions | General interpretation of the results |  |
| Trial registration | Registration number and name of trial register |  |
| Funding | Source of funding |  |
|  |  |  |

**REFERENCES**

1. Hopewell S, Clarke M, Moher D, Wager E, Middleton P, Altman DG, et al. CONSORT for reporting randomised trials in journal and conference abstracts. *Lancet* 2008, 371:281-283 [↑](#endnote-ref-1)
2. Hopewell S, Clarke M, Moher D, Wager E, Middleton P, Altman DG at al (2008) CONSORT for reporting randomized controlled trials in journal and conference abstracts: explanation and elaboration. *PLoS Med* 5(1): e20 [↑](#endnote-ref-2)
3. Ioannidis JP, Evans SJ, Gotzsche PC, O'Neill RT, Altman DG, Schulz K, Moher D. Better reporting of harms in randomized trials: an extension of the CONSORT statement. *Ann Intern Med* 2004; 141(10):781-788. [↑](#endnote-ref-3)
4. Relevant to Conference Abstracts [↑](#footnote-ref-1)
