# Supplementary material for: Independent and combined effects of improved water, sanitation, and hygiene (WASH) and improved complementary feeding on early neurodevelopment among children born to HIV-negative mothers in rural Zimbabwe: Substudy of a cluster-randomized trial
Source: PLoS Med. 2019 Mar 21;16(3):e1002766. doi: 10.1371/journal.pmed.1002766 (PMC6428259; doi:10.1371/journal.pmed.1002766)
Supplement: S1 Table — (DOCX) [file pmed.1002766.s003.docx]

**Supplementary Table 1: Baseline characteristics of mothers and infants who enrolled and did not enrol in the ECD substudy**

|  | Enrolled in ECD  1639 Mothers/1655 Infants | Not enrolled in ECD  2008 Mothers/2001 Infants | p Value |
| --- | --- | --- | --- |
| N |  |  |  |
| Mothers completing baseline visit^1^ | 1550 | 1854 |  |
| **Household characteristics** |  |  |  |
| Size, median (IQR) | 5 (3,6) | 5 (3,6) | 0.62 |
| Wealth Quintile^2^ |  |  | 0.36 |
| Lowest | 16.2 | 17.1 |  |
| Second | 18.5 | 17.4 |  |
| Middle | 19.8 | 18.0 |  |
| Fourth | 20.8 | 18.8 |  |
| Highest | 18.9 | 19.8 |  |
| ***Electricity*** |  |  |  |
| Power grid | 3.0 | 2.6 | 0.80 |
| Other power: |  |  | 0.79 |
| Generator | 3.3 | 3.3 |  |
| Solar | 69.0 | 68.0 |  |
| No electricity | 27.7 | 28.7 |  |
| ***Sanitation*** |  |  |  |
| Household members who openly defecate (all) | 47.9 | 49.5 | 0.07 |
| Household members who openly defecate (by age group): |  |  |  |
| 0-<3 years | 54.8 | 57.9 | 0.64 |
| 3-<6 years | 54.7 | 56.2 | 0.07 |
| 6-<18 years | 49.9 | 48.3 | 0.02 |
| 18-<70 years | 47.0 | 49.3 | 0.22 |
| >70 years | 30.3 | 32.5 | 0.58 |
| Any latrine at household | 37.6 | 36.6 | 0.52 |
| Improved latrine at household | 33.4 | 31.4 | 0.22 |
| Improved latrine with well-trodden path | 29.6 | 27.7 | 0.23 |
| Improved latrine with well-trodden path and not shared | 26.7 | 25.1 | 0.34 |
| ***Water*** |  |  |  |
| Main source of household drinking water is improved | 62.8 | 63.7 | 0.61 |
| Treat drinking water to make it safer | 13.8 | 11.8 | 0.10 |
| One-way walk time to fetch water (min); median (IQR) | 10 (5,15) | 10 (5, 20) | <0.001 |
| Per capita water volume collected in past 24 h (L); mean (SD) | 9.6 (9.4) | 9.7 (10.7) | <0.001 |
| ***Hygiene*** |  |  |  |
| Handwashing station at household | 10.5 | 9.2 | 0.21 |
| Handwashing station with water | 3.0 | 3.4 | 0.52 |
| Handwashing station with water and rubbing agent | 1.1 | 0.6 | 0.11 |
| Improved floor^3^ | 54.8 | 55.9 | 0.49 |
| Number of chickens; median (IQR) | 6 (2,10) | 6 (2,10) | 0.72 |
| Livestock in home | 39.0 | 36.1 | <0.001 |
| Feces observed in yard | 33.4 | 30.1 | 0.05 |
| ***Diet quality and food security*** |  |  |  |
| Household meets minimum Dietary Diversity Score^4^ | 41.5 | 38.4 | 0.08 |
| Coping strategies Index^5^; median (IQR) | 1 (0,7) | 1 (0,6) | 0.58 |
|  |  |  |  |
| **Maternal characteristics** |  |  |  |
| Age (y); mean (SD) | 26.5 (7.6) | 25.2 (6.8) | <0.001 |
| Height (cm); mean (SD) | 160.3 (6.0) | 160.1 (5.4) | <0.001 |
| MUAC (cm); mean (SD) | 26.5 (3.2) | 26.4 (3.2) | <0.001 |
| Completed schooling (y); mean (SD) | 9.6 (2.0) | 9.6 (2.2) | 0.39 |
| Parity; median (IQR) | 2 (1, 3) | 2 (1, 3) | 0.003 |
| Married | 95.8 | 95.1 | 0.36 |
| Employed | 9.4 | 7.9 | <0.001 |
| Religion: |  |  | 0.07 |
| Apostolic | 51.3 | 47.6 |  |
| Other Christian | 46.2 | 48.9 |  |
| Other | 2.6 | 3.5 |  |
|  |  |  |  |
| **Infant characteristics** |  |  |  |
| Female | 49.7 | 50.4 | 0.68 |
| Birth weight (kg) (SD) | 3.10 (0.52) | 3.12 (0.46) | <0.001 |
| Birth weight <2500 g | 8.3 | 8.0 | 0.84 |
| Institutional delivery | 89.3 | 89.5 | 0.82 |
| Vaginal delivery | 93.0 | 92.7 | 0.82 |

Values are %, unless stated.

^1^Baseline for mothers was 2 weeks after consent (~14 weeks gestation). Baseline for infants was at birth.

^2^Wealth index constructed as described in Yousafzai AK, Rasheed MA, Rizvi A, Armstrong R, Bhutta ZA. Effect of integrated responsive stimulation and nutrition interventions in the Lady Health Worker programme in Pakistan on child development, growth, and health outcomes: a cluster-randomised factorial effectiveness trial. Lancet. 2014. doi: 10.1016/S0140-6736(14)60455-4. PubMed PMID: 24947106.

^3^Improved floor defined as concrete, brick, cement or tile. Unimproved floor defined as mud, earth, sand or dung.

^4^Dietary Diversity Score households determined as described in Kochanska G, Murray KT, Harlan ET. Effortful control in early childhood: continuity and change, antecedents, and implications for social development. Developmental psychology. 2000;36(2):220-32. PubMed PMID: 10749079.

^5^Coping Strategies Index is a measure of household food insecurity, as described in Nampijja M, Apule B, Lule S, Akurut H, Muhangi L, Webb EL, et al. Effects of maternal worm infections and anthelminthic treatment during pregnancy on infant motor and neurocognitive functioning. Journal of the International Neuropsychological Society : JINS. 2012;18(6):1019-30. Epub 2012/11/20. doi: 10.1017/S1355617712000768. PubMed PMID: 23158229.
