# Supplementary material for: Independent and combined effects of improved water, sanitation, and hygiene (WASH) and improved complementary feeding on early neurodevelopment among children born to HIV-negative mothers in rural Zimbabwe: Substudy of a cluster-randomized trial
Source: PLoS Med. 2019 Mar 21;16(3):e1002766. doi: 10.1371/journal.pmed.1002766 (PMC6428259; doi:10.1371/journal.pmed.1002766)
Supplement: S1 Text — (DOCX) [file pmed.1002766.s004.docx]

**SUPPLEMENTARY APPENDIX**

**Independent and combined effects of improved water, sanitation and hygiene, and improved complementary feeding on early child development in rural Zimbabwe: substudy of a cluster-randomized trial**

Melissa J. Gladstone MD, Jaya Chandna MSc, Gwendoline Kandawasvika PhD, Robert Ntozini MPH, Florence D. Majo RGN, Naume V. Tavengwa MSW, Mduduzi N. N. Mbuya PhD, Goldberg T. Mangwadu MSc, Ancikaria Chigumira MSc, Cynthia M. Chasokela PhD, Lawrence H. Moulton PhD, Rebecca J. Stoltzfus PhD, Jean H. Humphrey ScD, and Andrew J. Prendergast DPhil for the SHINE Trial Team

Corresponding author: Dr. Melissa Gladstone, Department of Women and Children’s Health, Institute of Translational Medicine, University of Liverpool, Alder Hey Children’s NHS Foundation Trust, Eaton Road, L12 2AP, UK. Email: M.J.Gladstone@liverpool.ac.uk

**Contents**

Supplementary methods

a) Randomisation procedure p2-3

b) Changes in gestational age enrolment criteria p4

c) Further details of interventions p4-7

d) Validation and quality control of Early Child Development substudy p7

e) Definition of per protocol analysis p7-8

eTable 1: Baseline characteristics of mothers and infants who enrolled and did

not enrol in the early child development (ECD) sub-study p9-11

eTable 2: Per-protocol analysis: effect of WASH and IYCF interventions on

early child development at 24 months of age p12

**SUPPLEMENTARY METHODS**

**a) Randomisation procedure**

A highly constrained randomisation technique was used to allocate clusters (stratified by district) to treatments. We randomly selected 1000 allocations from among 5000 computer-generated allocations that balanced the 4 treatment arms on 14 parameters without pre-specified bounds. From the 1000, we randomly selected 10 for a public randomisation ceremony; Figure A. Each randomisation scheme divided the randomisation units into 4 groups of approximately 53 units. Each scheme’s corresponding colour-coded map was printed on a separate sheet and displayed at a public forum attended by all elected councillors from the study area, District and Provincial Administrators, and Ministry of Health and Child Care authorities. In their presence, 10 plastic balls (numbered 1-10) were placed in an opaque sack. A community representative selected one ball from the sack, thereby identifying which of the 10 numbered allocations would be used. Then, four balls (labelled A, B, C, and D) were placed in one sack, and four balls (labelled with the 4 treatment arms) were placed in a second sack. Representatives drew a ball from the first sack and a ball from the second sack, pairing a group of clusters with one of the four treatment arms, thereby mimicking a widely known World Cup draw procedure. This was repeated twice more to pair the next two groups of clusters with two more treatment arms. The remaining balls formed the final pairing. This second stage was included to provide an additional assurance of impartiality/randomness and a further opportunity for participation of the community leadership.


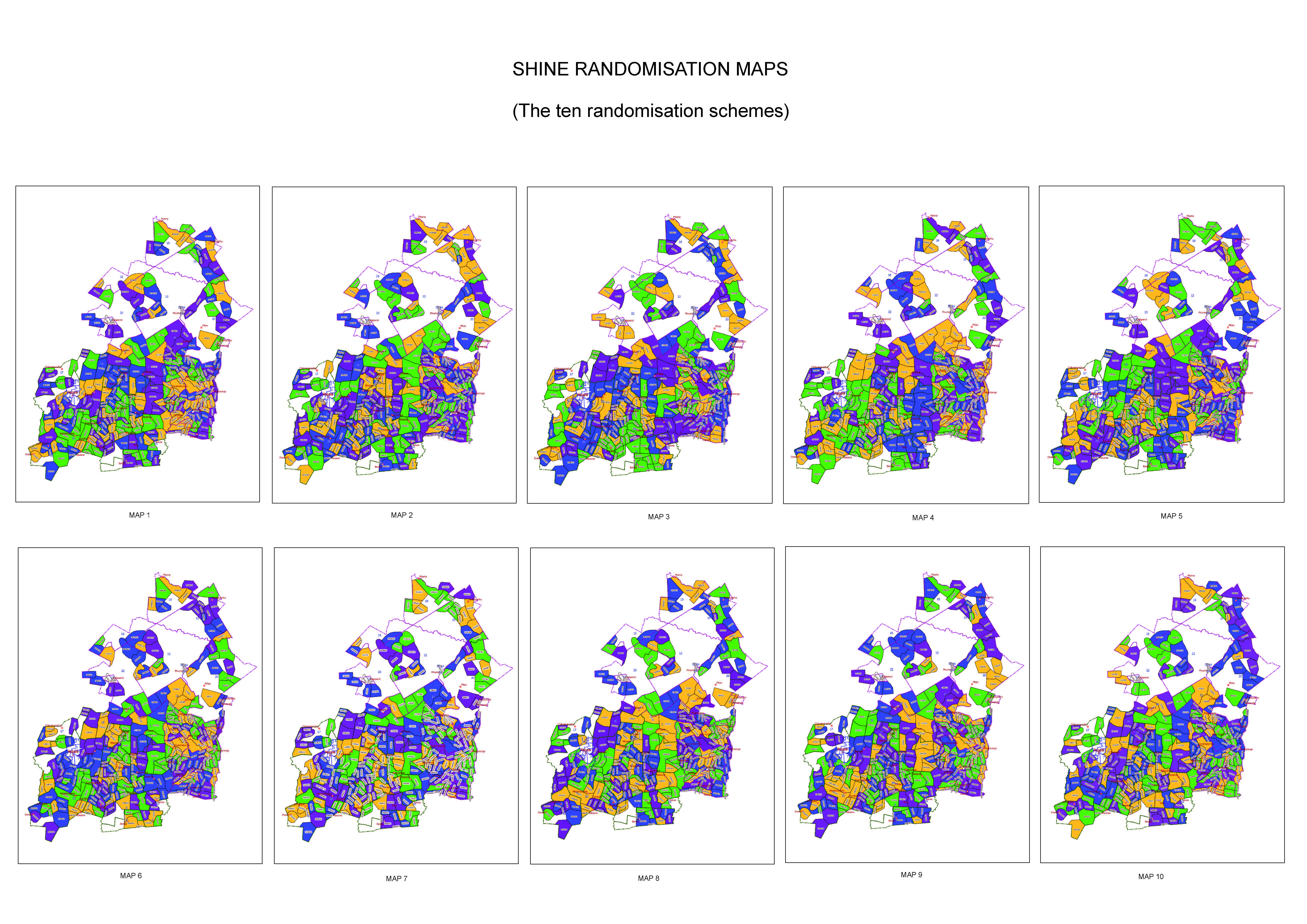


Legend: SOC IYCF WASH WASH+IYCF

**Figure A. Maps of ten SHINE randomization schemes**

**b) Changes in gestational age enrolment criteria**

The goal at the start of the trial was to recruit women between 10-14 weeks gestation, so women more than 14 weeks gestation were excluded. However, this cut-off was liberalised three times over the life of the trial to maximise recruitment because many women were excluded due to pregnancy that was >14 gestational weeks at screening. The upper limit of gestational age was therefore increased to 18 weeks (August 22, 2013), 24 weeks (January 3, 2014), and any time prior to parturition (October 20, 2014), through trial protocol amendments.

**c) Further details of interventions**

*Standard of Care (SOC) Intervention:* Village Health Workers were trained through the Ministry of Health and Child Care curriculum, which instructs VHWs to visit pregnant women and infants frequently, although the precise content or purpose of each visit is not specified. Consequently the SHINE SOC intervention was designed to standardize the number of visits (3 antenatal and 12 postnatal visits) and the content of primary health care messages across treatment arms. Four of these visits promoted exclusive breastfeeding (EBF) from birth to 6 months using modules designed to overcome contextual barriers identified in formative work. Other SOC modules include prevention of mother-to-child HIV transmission (PMTCT), antenatal care, hospital-based delivery, family planning and immunizations.

*WASH Intervention:* Within 6 weeks of enrolment (~20 weeks gestation) into the WASH and WASH+IYCF arms of the trial, a Blair Ventilated Improved Pit (VIP) Latrine was constructed at the participant’s household and two ‘Tippy Tap’ hand-washing stations (locally manufactured, and adapting the model piloted by the Kenya WASH Benefits trial) were installed near the latrine and kitchen; Figure B.


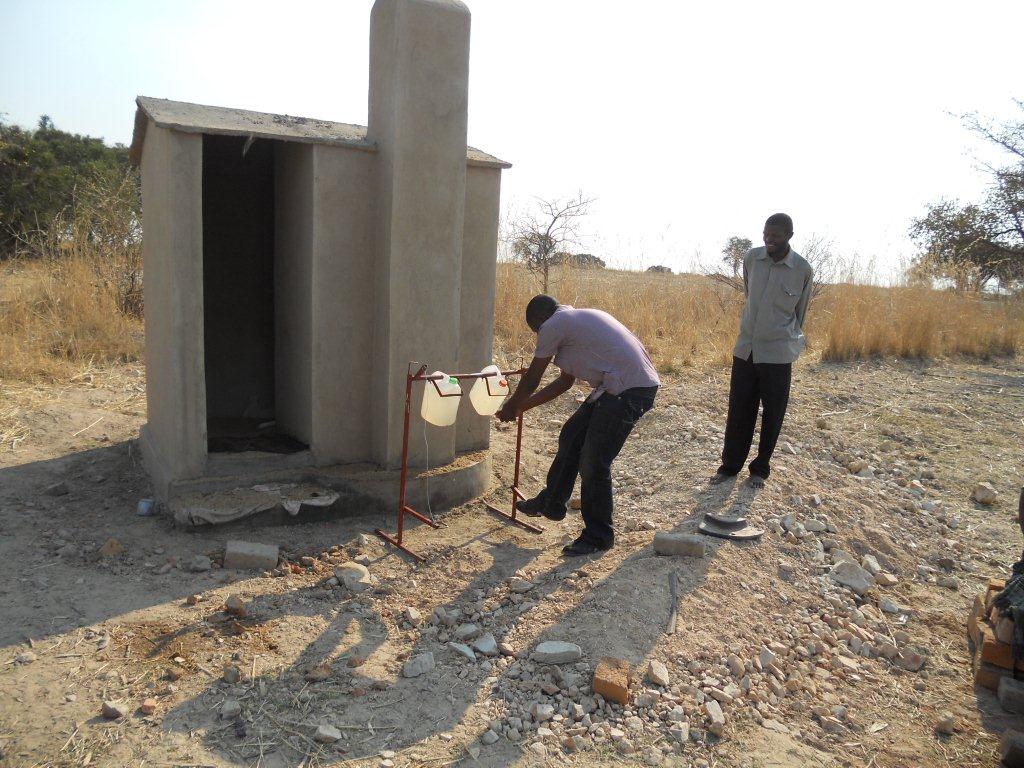


**Figure B: Blair VIP latrine and Tippy Tap**

WASH Modules 1 (delivered at 24 gestational weeks) and 2 (32 gestational weeks) promoted safe disposal of faeces, and hand-washing with soap after faecal contact and before food preparation and eating, respectively. Our intention was for the baby to be born into a household in which latrine use and household hand-washing behaviours were normalised and habitual. WASH Module 3 (protecting babies from faecal ingestion during exploratory play) was delivered when the baby was 2 months old; a washable 2.8m x 3.0m locally manufactured mat and plastic play yard (North States, Minneapolis MN) were provided at 2 months and 6 months, respectively; Figure C.
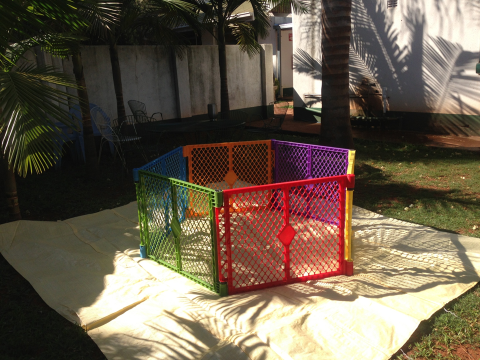


**Figure C: SHINE mat and play yard**

WASH Module 4 (treat all drinking water given to babies after 6 months of EBF) was delivered at 4 months of age, along with point-of-use chlorination (WaterGuard: a dilute sodium hypochlorite solution, manufactured locally by Nelspot). Liquid soap and Water Guard were regularly replenished from time of introduction (Module 2 and 5, respectively) until the infant was 18 months old. WASH Module 5, delivered at 5 months of age, stressed the importance of freshly preparing or fully reheating all foods fed to infants. A review module was delivered at 12 months.

*IYCF Intervention:* IYCF Module 1 (delivered at 5 months) linked good infant feeding to child growth, health, and intelligence. IYCF Module 2 (6 months) promoted feeding nutrient-dense food, including 20 g per day of the lipid-based nutrient supplement (LiNS) developed by the International Lipid-Based Nutrients Supplements Project, provided monthly when the baby was 6 to 18 months of age. Module 3 (7 months) was a participatory cooking demonstration in which any available household food was prepared and fed to the baby, stressing three messages from formative research: 1) an infant can eat any food that an adult eats; 2) food should be ground so that the infant can swallow and digest it; 3) food that is locally available is important for the infant. Module 4 (8 months) promoted responsive feeding during illness, Module 5 (9 months) promoted diet diversity, and a review module was delivered at 12 months.

**d) Validation and quality control of Early Child Development substudy**

Supportive supervision was provided at least monthly for each nurse by the study psychologist (JC). Every 6 months, nurses conducted an ECD assessment which was observed and double-marked by JC. If percentage agreement was < 85%, the nurse was retrained and retested until achieving 85% before testing any further study children. Inter-class correlation between nurses for the same child was high: MDAT 0.88 (95% CI: 0.82 to 0.94); McArthur Bates 0.94 (95% CI: 0.90 to 0.96); A-not-B 0.85 (95% CI: 0.80 to 0.90) and self-control 0.80 (95% CI: 0.76 to 0.85). A 10% subsample of assessments were video-recorded and reviewed by the study psychologist (JC) and a paediatrician with advanced training in child neurodevelopment and Shona language proficiency (GK). Percentage agreement on recorded assessments was 93% for MDAT fine motor, 90% for MDAT language, 97% for A-not-B and 91% for the self-control task.

**e) Definition of per protocol analysis**

Secondary modified per protocol analyses were conducted with these restrictions:

WASH intervention

Higher Fidelity:  received all 5 WASH modules and 12-month review

Lower Fidelity:  received less than all 5 WASH modules and 12-month review

IYCF intervention

Higher Fidelity:  received all 5 IYCF modules and 12-month review

Lower Fidelity:  received less than all 5 IYCF modules and 12-month review

**eTable 1: Baseline characteristics of mothers and infants who enrolled and did not enrol in the early child development (ECD) sub-study**

| **Baseline characteristic^a^** | **Enrolled in ECD** | **Not enrolled in ECD** | ***P* value** |
| --- | --- | --- | --- |
| Woman assessed, N | 1639 | 2008 |  |
| Infants assessed, N | 1655 | 2001 |  |
| Mothers completing baseline visit , N | 1550 | 1854 |  |
| **Household characteristics** |  |  |  |
| Size, median (IQR) | 5 (3,6) | 5 (3,6) | 0.690 |
| Wealth Quintile^b^ |  |  | 0.305 |
| Lowest | 16.1 | 17.4 |  |
| Second | 18.8 | 16.9 |  |
| Middle | 19.8 | 18.3 |  |
| Fourth | 20.3 | 18.6 |  |
| Highest | 19.2 | 19.9 |  |
| ***Electricity*** |  |  |  |
| Connected to power grid | 3.0 | 2.6 | 0.803 |
| Use other power source: |  |  |  |
| Generator | 3.3 | 3.3 | 0.785 |
| Solar | 69.0 | 68.0 |  |
| No electricity | 27.7 | 28.7 |  |
| ***Sanitation*** |  |  |  |
| Household members who openly defecate (all) | 48.4 | 51.0 | 0.054 |
| Household members who openly defecate (by age group): |  |  |  |
| 0-<3 years | 59.6 | 65.2 | 0.399 |
| 3-<6 years | 51.9 | 56.7 | 0.054 |
| 6-<18 years | 49.8 | 46.2 | 0.024 |
| 18-<70 years | 47.3 | 52.2 | 0.039 |
| >70 years | 50.0 | 40.9 | 0.940 |
| Any latrine at household | 37.6 | 36.6 | 0.519 |
| Improved latrine at household | 33.4 | 31.4 | 0.221 |
| Improved latrine with well-trodden path | 29.6 | 27.7 | 0.229 |
| Improved latrine with well-trodden path and not shared | 26.7 | 25.2 | 0.341 |
| ***Water*** |  |  |  |
| Main source of household drinking water is improved | 62.8 | 63.7 | 0.613 |
| Treat drinking water to make it safer | 13.8 | 11.8 | 0.095 |
| One-way walk time to fetch water, minutes; median (IQR) | 10 (5,15) | 10 (5, 20) | 0.001 |
| Per capita water volume collected in past 24 h, L; mean (SD) | 9.6 (9.4) | 9.7 (10.7) | <0.0001 |
| ***Hygiene*** |  |  |  |
| Handwashing station at household | 9.4 | 8.4 | 0.327 |
| Handwashing station with water | 3.0 | 3.3 | 0.641 |
| Handwashing station with water and rubbing agent | 1.0 | 0.6 | 0.118 |
| Improved floor | 54.8 | 55.9 | 0.494 |
| Number of chickens; median (IQR) | 6 (2,10) | 6 (2,10) | 0.716 |
| Livestock in home | 39.5 | 36.7 | <0.0001 |
| Feces observed in yard | 33.2 | 30.2 | 0.074 |
| ***Diet quality and food security*** |  |  |  |
| Household meets minimum Dietary Diversity Score^c^ | 41.5 | 38.4 | 0.081 |
| Coping strategies Index^d^; median (IQR) | 1 (0,7) | 1 (0,6) | 0.578 |
|  |  |  |  |
| **Maternal characteristics** |  |  |  |
| Age, years; mean (SD) | 26.5 (7.6) | 25.2 (6.8) | <0.0001 |
| Height, cm; mean (SD) | 160.3 (6.0) | 160.1 (5.4) | <0.0001 |
| MUAC, cm; mean (SD) | 26.5 (3.2) | 26.4 (3.2) | <0.0001 |
| Completed schooling, years; mean (SD) | 9.6 (2.0) | 9.6 (2.2) | 0.389 |
| Parity; median (IQR) | 2 (1, 3) | 2 (1, 3) | 0.003 |
| Married | 95.8 | 95.1 | 0.361 |
| Employed | 9.4 | 7.9 | <0.0001 |
| Religion: |  |  |  |
| Apostolic | 51.3 | 47.6 | 0.069 |
| Other Christian | 46.2 | 48.9 |  |
| Other | 2.6 | 3.5 |  |
|  |  |  |  |
| **Infant characteristics** |  |  |  |
| Female | 50.3 | 49.6 | 0.679 |
| Birth weight, kg; mean (SD) | 3.10 (0.51) | 3.12 (0.46) | <0.0001 |
| Birth weight <2500 g | 8.3 | 8.0 | 0.791 |
| Institutional delivery | 89.3 | 89.5 | 0.819 |
| Vaginal delivery | 93.0 | 92.7 | 0.230 |

Values are %, unless stated.

^a^ Baseline for mothers was 2 weeks after consent (~14 weeks gestation). Baseline for infants was at birth.

^b^ Chasekwa B, Maluccio JA, Ntozini R, Moulton LH, Wu F, Smith LE, et al. Measuring wealth in rural communities: Lessons from the Sanitation, Hygiene, Infant Nutrition Efficacy (SHINE) trial. PLoS ONE. 2018; 13(6): e0199393.3Improved floor defined as concrete, brick, cement or tile. Unimproved floor defined as mud, earth, sand or dung.

^c^ FAO, FHI 360. Minimum Dietary Diversity for Women: A Guide for Measurement. Rome: FAO. 2016

^d^ Coping Strategies Index is a measure of household food insecurity, described in Maxwell D, Watkins B, Wheeler R, Collins G. The Coping Strategy Index: A tool for rapid measurement of household food security and the impact of food aid programs in humanitarian emergencies. CARE and WFP, Nairobi. 2003.

**Supplementary Table 1: Comparison of baseline characteristics across 3 groups of infants: Infants who completed the 18 month visit but were not eligible for the ECD Substudy; Infants who were eligible for ECD but were not enrolled into ECD; and Infants who were eligible for ECD and were enrolled into ECD**

| **Baseline characteristic^a^** | **Infants who completed the 18 month visit but were not eligible for the ECD Substudy** | **Infants who were eligible for ECD but were not enrolled into ECD Substudy** | **Infants who were eligible for ECD and were enrolled into ECD Substudy** | **P-value** |
| --- | --- | --- | --- | --- |
| Woman assessed, N | 1076 |  |  |  |
| Infants assessed, N | 1085 |  |  |  |
| Women completing baseline visit, N | 846 |  |  |  |
| **Household characteristics** |  |  |  |  |
| Size; median (IQR) | 4 (3,6) | 5 (3,6) | 5 (3,6) | 0.57 |
| Wealth Quintile^c^ |  |  |  |  |
| Lowest | 17.5 | 17.4 | 16.1 | 0.43 |
| Second | 17.7 | 15.9 | 18.8 |  |
| Middle | 19.7 | 16.8 | 19.8 |  |
| Fourth | 18.4 | 19.0 | 20.3 |  |
| Highest | 19.2 | 20.7 | 19.3 |  |
| ***Electricity*** |  |  |  |  |
| Power grid | 2.4 | 2.9 | 3.3 | 0.93 |
| Other power: |  |  |  |  |
| Generator | 3.0 | 3.6 | 3.3 | 0.13 |
| Solar | 65.9 | 70.5 | 69.1 |  |
| No electricity | 31.1 | 26.0 | 27.6 |  |
| ***Sanitation*** |  |  |  |  |
| Household members who openly defecate (all) | 53.4 | 48.2 | 48.4 | <0.0001 |
| Household members who openly defecate (by age group): |  |  |  |  |
| 0-<3 years | 66.7 | 62.5 | 59.6 | 0.46 |
| 3-<6 years | 57.1 | 57.5 | 52.0 | <0.0001 |
| 6-<18 years | 49.8 | 42.2 | 49.5 | 0.0005 |
| 18-<70 years | 54.1 | 49.9 | 47.3 | 0.056 |
| >70 years | 25.0 | 60.0 | 50.0 | <0.0001 |
| Any latrine at household | 36.1 | 37.2 | 37.7 | 0.73 |
| Improved latrine at household | 30.3 | 32.7 | 33.5 | 0.30 |
| Improved latrine with well-trodden path | 26.6 | 29.0 | 29.6 | 0.26 |
| Improved latrine with well-trodden path and not shared | 23.9 | 26.7 | 26.8 | 0.27 |
| ***Water*** |  |  |  |  |
| Main source of household drinking water is improved | 64.47 | 63.1 | 62.7 | 0.74 |
| Treat drinking water to make it safer | 11.8 | 11.8 | 13.8 | 0.26 |
| One-way walk time to fetch water (min); median (IQR) | 10 (5, 20) | 10 (5, 20) | 10 (5, 15) | 0.0005 |
| Per capita water volume collected past 24h (L); mean (SD) | 9.7 (8.6) | 9.8 (11.0) | 9.6 (9.4) | <0.0001 |
| ***Hygiene*** |  |  |  |  |
| Handwashing station at household | 5.6 | 11.5 | 9.4 | <0.0001 |
| Handwashing station with water | 2.4 | 4.3 | 3.0 | 0.04 |
| Handwashing station with water and rubbing agent | 0.7 | 0.5 | 1.0 | 0.27 |
| Improved floor^d^ | 56.0 | 55.9 | 54.8 | 0.80 |
| Number of chickens; median (IQR) | 6 (2, 10) | 6 (2, 10) | 6 (2, 10) | 0.53 |
| Livestock observed inside the house | 35.5 | 38.2 | 39.6 | <0.0001 |
| Feces observed in the yard | 30.2 | 30.1 | 33.2 | 0.20 |
| ***Diet quality and food security*** |  |  |  |  |
| Household meets minimum Dietary Diversity Score^e^ | 38.8 | 37.5 | 41.7 | 0.14 |
| Coping strategies Index^f^_;_ median (IQR) | 1 (0, 8) | 0 (0, 5) | 1 (0, 7) | 0.0003 |
|  |  |  |  |  |
| **Maternal characteristics** |  |  |  |  |
| Age (y); mean (SD) | 26.1 (6.5) | 24.1 (6.3) | 26.5 (7.7) | <0.0001 |
| Height (cm); mean (SD) | 160.2 (5.9) | 159.8 (5.4) | 160.3 (6) | <0.0001 |
| MUAC (cm); mean (SD) | 26.7 (3.2) | 26.0 (3.1) | 26.5 (3.2) | <0.0001 |
| Completed schooling (y); mean (SD) | 9.6 (2.0) | 9.6 (1.9) | 9.6 (2.0) | 0.088 |
| Parity; median (IQR) | 1 (0, 2) | 1 (0, 2) | 2 (1, 3) | <0.0001 |
| Married | 97.1 | 92.8 | 95.8 | 0.0002 |
| Employed | 7.6 | 8.2 | 9.4 | <0.0001 |
| Religion: |  |  |  |  |
| Apostolic | 47.0 | 48.3 | 51.3 | 0.089 |
| Other Christian (Pentecostal, Catholic, Other Christian) | 48.8 | 48.9 | 46.2 |  |
| Other religion (Muslim and other) | 4.2 | 2.8 | 2.6 |  |
| Maternal Capabilities^g^: |  |  |  |  |
| Gender norms and attitudes; mean (SD) | 1.95 (0.89) | 2.35 (0.83) | 2.25 (1.00) | <0.0001 |
| Perceived social support; mean (SD) | 3.5 (0.71) | 3.62 (0.76) | 3.61 (0.67) | <0.0001 |
| Perceived physical health; mean (SD) | 3.35 (1.01) | 3.46 (1.09) | 3.41 (0.96) | <0.0001 |
| Mothering self-efficacy; mean (SD) | 3.97 (0.42) | 3.92 (0.43) | 3.98 (0.40) | <0.0001 |
| Perceived time stress; mean (SD) | 2.71 (0.73) | 2.58 (0.80) | 2.66 (0.86) | <0.0001 |
| Decision making autonomy; median (IQR) | 5 (3, 5) | 5 (4, 5) | 5 (4, 5) | 0.003 |
| **Infant characteristics** |  |  |  |  |
| Female | 48.6 | 50.7 | 50.3 | 0.51 |
| Birth weight (kg); mean (SD) | 3.16 (0.44) | 3.07 (0.47) | 3.11 (0.51) | <0.0001 |
| Birth weight <2500 g | 5.4 | 10.9 | 8.2 | <0.0001 |
| Institutional delivery | 89.0 | 90.2 | 89.3 | 0.66 |
| Vaginal delivery | 92.9 | 92.5 | 93.1 | 0.47 |

Values are % unless otherwise stated

^a^ Baseline for mothers was 2 weeks after consent (~14 weeks gestation). Baseline for infants was at birth.

^b^ SOC=Standard of Care; IYCF = Infant and Young Child Feeding; WASH = Water, sanitation and hygiene

^c^ Chasekwa B, Maluccio JA, Ntozini R, Moulton LH, Wu F, Smith LE, et al. Measuring wealth in rural communities: Lessons from the Sanitation, Hygiene, Infant Nutrition Efficacy (SHINE) trial. PLoS ONE. 2018; 13(6): e0199393.

^d^Improved floor defined as concrete, brick, cement or tile. Unimproved floor defined as mud, earth, sand or dung.

^e^ FAO, FHI 360. Minimum Dietary Diversity for Women: A Guide for Measurement. Rome: FAO. 2016.

^f^Coping Strategies Index is a measure of household food insecurity, as described in Maxwell D, Watkins B, Wheeler R, Collins G. The Coping Strategy Index: A tool for rapid measurement of household food security and the impact of food aid programs in humanitarian emergencies. CARE and WFP, Nairobi. 2003.

^f^ Need ref for mat caps

**eTable 2. Effects of WASH and IYCF interventions on early child development at 24 months of age among participants with high-fidelity^a^ delivery of intervention**

| **Primary continuous outcomes** | **Effects by arm** | | | |  | | **Main Effects combining arms** | | | | | | |
| --- | --- | --- | --- | --- | --- | --- | --- | --- | --- | --- | --- | --- | --- |
|  | **Treatment group** | **N** | **Mean (SD)** |  | | **Treatment group** | | **N** | **Mean (SD)** | **Unadjusted Difference (95% CI)** | ***P* Value** | |  |
| **MDAT Total Score** | SOC | 291 | 92.6 (9.9) |  | | IYCF: no | | 635 | 92.1 (9.4) | 0.0 (ref) |  | |  |
|  | IYCF | 304 | 92.3 (8.6) |  | | IYCF: yes | | 675 | 93.0 (9.0) | 0.94 (-0.31, 2.20) | 0.140 | |  |
|  | WASH | 344 | 91.7 (9.0) |  | | WASH: no | | 595 | 92.4 (9.3) | 0.0 (ref) |  | |  |
|  | IYCF+WASH | 371 | 93.6 (9.2) |  | | WASH: yes | | 715 | 92.7 (9.2) | 0.46 (-79, 1.71) | 0.470 | |  |
| **MDAT Gross Motor** | SOC | 291 | 23.7 (3.2) |  | | IYCF: no | | 635 | 23.5 (3.1) | 0.0 (ref) |  | |  |
|  | IYCF | 304 | 23.6 (2.9) |  | | IYCF: yes | | 675 | 23.7 (3.2) | 0.21 (-0.18, 0.60) | 0.300 | |  |
|  | WASH | 344 | 23.3 (3.0) |  | | WASH: no | | 595 | 23.6 (3.1) | 0.0 (ref) |  | |  |
|  | IYCF+WASH | 371 | 23.9 (3.4) |  | | WASH: yes | | 715 | 23.6 (3.2) | -0.01 (-0.40, 0.39) | 0.974 | |  |
| **MDAT Fine Motor** | SOC | 291 | 23.3 (2.8) |  | | IYCF: no | | 635 | 23.2 (2.5) | 0.0 (ref) |  | |  |
|  | IYCF | 304 | 23.1 (2.7) |  | | IYCF: yes | | 675 | 23.4 (2.4) | -0.13 (-0.18, 0.44) | 0.401 | |  |
|  | WASH | 344 | 23.2 (2.2) |  | | WASH: no | | 595 | 23.3 (2.6) | 0.0 (ref) |  | |  |
|  | IYCF+WASH | 371 | 23.5 (2.3) |  | | WASH: yes | | 715 | 23.4 (2.3) | -0.17 (-0.14, 0.48) | 0.292 | |  |
| **MDAT Language** | SOC | 291 | 21.4 (4.4) |  | | IYCF: no | | 635 | 21.2 (4.2) | 0.0 (ref) |  | |  |
|  | IYCF | 304 | 21.4 (4.2) |  | | IYCF: yes | | 675 | 21.6 (4.2) | 0.44 (-0.16, 1.04) | 0.151 | |  |
|  | WASH | 344 | 21.1 (4.1) |  | | WASH: no | | 595 | 21.4 (4.3) | 0.0 (ref) |  | |  |
|  | IYCF+WASH | 371 | 21.8 (4.1) |  | | WASH: yes | | 715 | 21.4 (4.1) | 0.11 (-0.50, 0.71) | 0.728 | |  |
| **MDAT Social** | SOC | 291 | 24.2 (2.1) |  | | IYCF: no | | 635 | 24.2 (2.5) | 0.0 (ref) |  | |  |
|  | IYCF | 304 | 24.2 (2.1) |  | | IYCF: yes | | 675 | 24.3 (2.2) | 0.17 (-0.10, 0.45) | 0.214 | |  |
|  | WASH | 344 | 24.2 (2.7) |  | | WASH: no | | 595 | 24.2 (2.1) | 0.0 (ref) |  | |  |
|  | IYCF+WASH | 371 | 24.4 (2.2) |  | | WASH: yes | | 715 | 24.3 (2.5) | 0.14 (-0.13, 0.41) | 0.300 | |  |
| **McArthur Bates (CDI)** | SOC | 284 | 61.1 (19.0) |  | | IYCF: no | | 620 | 61.7 (18.7) | 0.0 (ref) |  | |  |
|  | IYCF | 294 | 61.1 (19.0) |  | | IYCF: yes | | 656 | 62.5 (18.8) | 1.06 (-1.26, 3.38) | 0.371 | |  |
|  | WASH | 336 | 62.2 (18.5) |  | | WASH: no | | 578 | 61.1 (19.0) | 0.0 (ref) |  | |  |
|  | IYCF+WASH | 362 | 63.6 (18.6) |  | | WASH: yes | | 698 | 62.9 (18.6) | 1.83 (-0.50, 4.16) | 0.124 | |  |
| **A Not B** | SOC | 273 | 7.8 (1.3) |  | | IYCF: no | | 587 | 7.8 (1.3) | 0.0 (ref) | |  |  |
|  | IYCF | 280 | 7.8 (1.4) |  | | IYCF: yes | | 628 | 7.8 (1.4) | 0.05 (-0.12, 0.22) | | 0.584 |  |
|  | WASH | 314 | 7.8 (1.3) |  | | WASH: no | | 553 | 7.8 (1.3) | 0.0 (ref) | |  |  |
|  | IYCF+WASH | 348 | 7.9 (1.4) |  | | WASH: yes | | 662 | 7.8 (1.4) | 0.02 (-0.15, 0.19) | | 0.584 |  |
| **Primary dichotomous outcomes** | **Treatment group** | **N** | **Children with self-control**  **%** |  | | **Treatment group** | | **N** | **Children with self-control**  **%** | **Unadjusted Relative Risk**  **(95% CI)** | ***P* Value** | |  |
| **Self-control Task (Hidden)** | SOC | 283 | 34.3 |  | | IYCF: no | | 619 | 36.3 | 1.00 (ref) | |  |  |
|  | IYCF | 299 | 34.4 |  | | IYCF: yes | | 669 | 35.1 | 0.96 (0.83, 1.11) | | 0.617 |  |
|  | WASH | 336 | 38.1 |  | | WASH: no | | 582 | 34.4 | 1.00 (ref) | |  |  |
|  | IYCF+WASH | 370 | 35.7 |  | | WASH: yes | | 706 | 36.8 | 1.07 (0.93, 1.24) | | 0.343 |  |
| **Self-control Task**  **(Unhidden)** | SOC | 278 | 52.5 |  | | IYCF: no | | 613 | 55.0 | 1.00 (ref) | |  |  |
|  | IYCF | 297 | 54.2 |  | | IYCF: yes | | 664 | 54.1 | 0.98 (0.89, 1.08) | | 0.656 |  |
|  | WASH | 335 | 57.0 |  | | WASH: no | | 575 | 53.4 | 1.00 (ref) | |  |  |
|  | IYCF+WASH | 367 | 54.0 |  | | WASH: yes | | 702 | 55.4 | 1.04 (0.95, 1.15) | | 0.397 |  |

^a^ High fidelity was defined as participants who received all 5 IYCF modules and 12-month review if they were in the IYCF arms, or received all 5 WASH modules and 12-month review if they were in the WASH arms. Participants who received high fidelity intervention were: SOC=78% (291/374), IYCF=77% (304/393), WASH=84% (344/408), and WASH+IYCF=84% (371/440)
